# On Applicability of the Relaxation Spectrum of Fractional Maxwell Model to Description of Unimodal Relaxation Spectra of Polymers

**DOI:** 10.3390/polym15173552

**Published:** 2023-08-26

**Authors:** Anna Stankiewicz

**Affiliations:** Department of Technology Fundamentals, Faculty of Production Engineering, University of Life Sciences in Lublin, 20-612 Lublin, Poland; anna.m.stankiewicz@gmail.com

**Keywords:** viscoelasticity, relaxation spectrum, linear relaxation modulus, fractional Maxwell model, spectrum monotonicity, local spectrum extrema, BSW spectrum

## Abstract

The relaxation time and frequency spectra are vital for constitutive models and for insight into the viscoelastic properties of polymers, since, from the spectra, other material functions used to describe rheological properties of various polymers can be uniquely determined. In recent decades the non-integer order differential equations have attracted interest in the description of time-dependent processes concerning relaxation phenomena. The fractional Maxwell model (FMM) is probably the most known rheological model of non-integer order. However, the FMM spectrum has not yet been studied and used to describe rheological materials. Therefore, the goal of the present paper was to study the applicability of the relaxation spectrum of FMM to the description of the relaxation spectra of polymers. Based on the known integral representation of the Mittag-Leffler two-parameter function, analytical formulas describing relaxation time and frequency spectra of FMM model were derived. Monotonicity of the spectra was analyzed and asymptotic properties were established. Relaxation frequency spectrum grows for large frequencies with a positive power law, while the relaxation time spectrum decays for large times with a negative power of time. Necessary and sufficient conditions for the existence of the local extrema of the relaxation spectra were derived in the form of two trigonometric inequalities. A simple procedure for checking the existence or absence of the spectra extrema was developed. Direct analytical formulas for the local extrema, minima, and maxima are given in terms of model fractional and viscoelastic parameters. The fractional model parameters, non-integer orders of the stress and strain derivatives of FMM uniquely determine the existence of the spectrum extrema. However, the viscoelastic parameters of the FMM, elastic modulus, and relaxation time affect the maxima and minima of the relaxation spectra and the values of their local peaks. The influence of model parameters on their local extrema was examined. Next, the applicability of the continuous–discrete spectrum of FMM to describe Baumgaertel, Schausberger and Winter (BSW) and unimodal Gauss-like relaxation spectra, commonly used to describe rheological properties of various polymers, was examined. Numerical experiments have shown that by respective choice of the FMM parameters, in particular by respective choice of the orders of fractional derivatives of the stress and strain, a good fit for the relaxation modulus experiment data was obtained for polymers characterized both by BSW and Gauss-like relaxation spectra. As a result, a good approximation of the real spectra was reached. Thus, the viscoelastic relaxation spectrum of FMM, due to the availability of the two extra degrees of freedom (non-integer orders of the stress and strain derivatives), provides deep insights into the complex behavior of polymers and can be applied for a wide class of polymers with unimodal relaxation spectra.

## 1. Introduction

For several decades, apart from the classical integer-order differential models, fractional order rheological models have been widely adopted to describe the combined elastic and viscous properties of various polymers. In fractional calculus the operations of integration and differentiation are of non-integer (fractional) order [1]. Theoretical studies have been devoted to the study of fractional-order rheological models, e.g., [2,3,4,5,6] and their application to the description of polymers, for example, poly-isobutylene [4], polyurea and PET [6], shape memory polymers [7], amorphous polymers [8], and flax fiber reinforced polymer [9].

The viscoelastic behavior of polymers varies depending on the type of polymer [10,11], therefore different fractional models have been and are still being developed. Exponential relaxation is often modeled by classic or fractional Maxwell models [2,3]. When the Debye decays show deviations from Maxwell models, solutions can be approximated by the exponential stretched Kohlrausch–Williams–Watts (KWW) model [12,13]. To approximate non-exponential relaxation, inverse power-laws were also used [14,15,16,17]. Simultaneously, the relaxation processes described by fractional Maxwell model can be fitted by asymptotic power-law for small and large times [3,18], while the KWW model fits fractional Maxwell model for short times [3]. Fractional viscoelasticity, a new formalism introduced for mathematical modeling of rheological materials [14], appears to be a solid tool to describe the relaxation processes in polymers exhibiting both exponential and non-exponential type. Fractional order models have gained research interest due to their improved flexibility and better adjustment of their time-dependent properties, compared to those offered by their classic, integer order, counterparts.

Fractional Maxwell and Kelvin–Voight models are probably the most known fractional rheological models, similarly as for integer order differential viscoelastic models [2,4,5]. However, a deep insight into the complex behavior of polymers was also provided by the viscoelastic relaxation spectrum [11,19,20]. The relaxation spectrum is vital for constitutive models and for the insight into the properties of a viscoelastic material, since from the relaxation spectrum other material functions used to describe rheological properties of the material can be uniquely determined. Therefore, the spectrum is commonly used to describe, analyze, compare, and improve the mechanical properties of polymers [20,21,22,23,24].

However, there are no papers concerning the relaxation spectra of the fractional order viscoelastic models, even the fractional Maxwell model (FMM). Although Mainardi [4,25] and Mainardi and Spada [5] gave a spectral representation of the product of the Mittag-Leffler one-parameter function and power of time that provides the solution to the fractional Maxwell model with identical orders of the stress and strain derivatives, it can be directly related to the niche the definition of the relaxation spectrum as the inverse Laplace transform of the linear relaxation modulus. The possibility of using the FMM relaxation spectrum for modelling the relaxation spectra of polymers has not been studied so far. Thus, the determination and investigation of the relaxation spectrum of FMM is still an open issue.

Therefore, determination of the relaxation spectra of FMM, their analysis, and studying the applicability of these spectra to description of the relaxation spectra of polymers were the goals of the present paper.

First, starting from the known integral representation of the Mittag-Leffler two-parameter function, the relaxation time and frequency spectra of the fractional Maxwell model were derived in the form of direct analytical formulas. Next, the monotonicity of the spectra was analyzed, and asymptotic properties were established. Necessary and sufficient conditions for the existence of the local extrema of the relaxation spectra were derived in the form of two trigonometric inequalities. Also, some necessary conditions for the local extrema existence were given in the form of simpler inequalities. A fast procedure for checking the existence or not of the spectra extrema was presented based on the necessary and sufficient extreme conditions. Direct analytical formulas for the local extrema, minima and maxima were given in terms of model fractional and viscoelastic parameters. The fractional model parameters, namely non-integer orders of the stress and strain derivatives of FMM, uniquely determine the existence of the spectrum extrema. However, the local maxima and minima also depend on the relaxation time of FMM, and the values of the local extrema are affected by the elastic modulus of FMM.

Next, the applicability of the continuous spectrum of FMM to describe Baumgaertel, Schausberger, and Winter (BSW) [26,27] and Gauss-like relaxation spectra was examined. The BSW spectrum is often used to describe rheological properties of various polymers; for example, polydisperse polymer melts [28,29], polymethylmethacrylate (PMMA) and polybutadiene (PBD) [30], and polymer pelts [31]. Gauss-like distributions were used to describe rheological properties of, e.g., poly(methyl methacrylate) [32], polyethylene [33], native starch gels [34], polyacrylamide gels [35], and carboxymethylcellulose [36].

Numerical studies were conducted, and a good approximation of the real spectra was reached. Thus, the viscoelastic relaxation spectrum of FMM can be applied for a wide class of polymers with unimodal relaxation spectra. The applicability of the relaxation spectra of fractional order viscoelastic models to the description of multimodal spectra will be the subject of future research, with particular attention to bimodal spectra.

In Appendix A, the proofs and derivations of some mathematical formulas are given to increase the clarity of the article.

## 2. Materials and Methods

### 2.1. Maxwell Model

**Figure 1:**
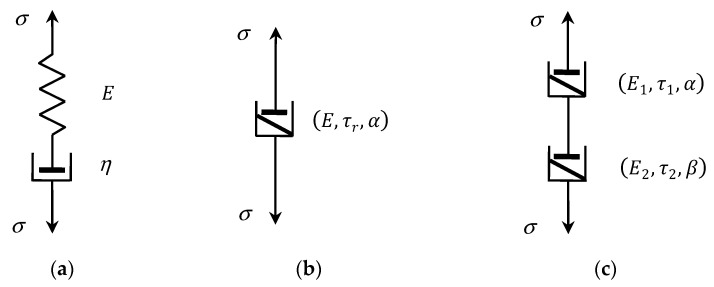
(**a**) Classic Maxwell model; (**b**) fractional Scott-Blair model of a non-integer positive order α; (**c**) fractional Maxwell model; elastic modulus E, $E_{1}$, $E_{2}$, viscosity η, relaxation times $\tau_{r}$, $\tau_{1}$, $\tau_{2}$.

Classic viscoelastic Maxwell model is the arrangement of ideal spring in a series with a dashpot (see Figure 1a) described by the first order differential equation [11,37]:

(1) $$\frac{d\sigma \left(t\right)}{dt}+\frac{E}{\eta }\sigma \left(t\right)=E\frac{d\varepsilon \left(t\right)}{dt},$$

where σ(t) and ε(t) denote the stress and strain, respectively, E is the elastic modulus of the spring, and η means the viscosity of the dashpot. Assuming unit-step strain ε(t) the uniaxial stress response of Maxwell model (1), i.e., the time-dependent relaxation modulus σ(t)=G(t), has exponential type given by [11,37]

G(t)=Ee−tτr,

with the relaxation time $\tau_{r}=\eta /E$.

### 2.2. Elementary Fractional Scott-Blair Model

Elementary fractional Scott-Blair model [2,4,38] is described by the fractional differential equation:

(2) $$\sigma \left(t\right)=E\tau_{r}^{\alpha }\frac{d^{\alpha }\varepsilon \left(t\right)}{dt^{\alpha }},$$

where α is non-integer positive order of fractional derivative of the strain ε(t). Here, $\frac{d^{\alpha }}{dt^{\alpha }}f\left(x\right)=D_{t}^{\alpha }f\left(x\right)$ means the fractional derivative operator in the sense of Caputo’s fractional derivative of a function f(x) of non-integer order α with respect to variable t and with a starting point at t=0, which is defined by [1,4]:

$$D_{t}^{\alpha }f\left(t\right)=\frac{1}{\Gamma \left(n-\alpha \right)}\int_{0}^{t}{\left(t-1\right)}^{n-\alpha -1}\frac{d^{n}}{dt^{n}}f\left(t\right)dt,$$

where n−1<α<n and Γ(n) is Euler’s gamma function [1] (Equation (A.1.1)).

Assuming unit-step strain ε(t), the uniaxial stress response G(t) of fractional element (2) is given by [2,4,38]:

(3) $$G\left(t\right)=\frac{E}{\Gamma \left(1-\alpha \right)}{\left(\frac{t}{\tau_{r}}\right)}^{-\alpha },$$

i.e., is represented by power of time law.

The fractional Scott-Blair model is an intermediate model between ideal spring σ(t)=Eε(t) and the Newton’s model $\sigma (t)=\eta \frac{d\varepsilon (t)}{dt}$ of ideal fluids represented by means of an ideal dashpot of viscosity η. The elementary fractional element (2) is uniquely described by three parameters $\left(E,\tau_{r},\alpha \right)$, as shown in Figure 1b. The first material described in 1944 by Scott-Blair and Veinoglou [39] using the fractional inverse power model (3) was bitumen. Following that, the inverse power-laws with various exponents were used for modelling many relaxation processes which have been reviewed by Bonfanti et al. [14]. Winter and Chambon [40] derived a power-type relaxation modulus with an exponent of −1/2 for cross-linking polymers at their gel point, which were used to analyze polydimethylsiloxane gel data. Likhtman and McLeish [15], studying the stress relaxation dynamics of linear entangled polymers (polystyrene and polybutadiene), dismissed the BSW dynamics and applied multiplicative exponential-power-laws models. Similar models were applied by Kapnistos et al. [16] for modelling the stress relaxation for entangled ring polymers which have a characteristic entanglement plateau.

### 2.3. Fractional Maxwell Model

Connecting in a series, by analogy to classic Maxwell model, two elementary fractional Scott-Blair elements $\left(E_{1},\tau_{1},\alpha \right)$ and $\left(E_{2},\tau_{2},\beta \right)$, see Figure 1c, we obtained fractional Maxwell model (FMM) described by the fractional differential equation [2,4,38]:

(4) $$\tau_{r}^{\alpha -\beta }\frac{d^{\alpha -\beta }\sigma \left(t\right)}{dt^{\alpha -\beta }}+\sigma \left(t\right)=E\tau_{r}^{\alpha }\frac{d^{\alpha }\varepsilon \left(t\right)}{dt^{\alpha }},$$

where the parameters E and $\tau_{r}$ are uniquely defined by the model components parameters according to [18]:

$$\tau_{r}={\left[\frac{E_{1}{\left(\tau_{1}\right)}^{\alpha }}{E_{2}{\left(\tau_{2}\right)}^{\beta }}\right]}^{\frac{1}{\alpha -\beta }},$$

$$E={\left[\frac{{\left(E_{1}\tau_{1}\right)}^{-\beta }{\left(\tau_{1}\right)}^{\alpha \left(1-\alpha \right)}}{{\left[E_{2}{\left(\tau_{2}\right)}^{\beta }\right]}^{-\alpha }}\right]}^{\frac{1}{\alpha -\beta }}.$$

For details of model (4) construction see, for example, [2,18]. The relaxation modulus G(t) of FMM (4) is known for an arbitrary 0≤ β≤α≤1 and given by the formula [2,4,5]:

(5) $$G\left(t\right)=E{\left(\frac{t}{\tau_{r}}\right)}^{-\beta }E_{\alpha -\beta ,1-\beta }\left(-{\left(\frac{t}{\tau_{r}}\right)}^{\alpha -\beta }\right),$$

where $E_{\kappa ,\mu }\left(x\right)$ is the generalized two-parameter Mittag-Leffler function defined by series representation, convergent in the whole z-complex plane [1,2]:

(6) $$E_{\kappa ,\mu }\left(x\right)=\sum_{n=0}^{\infty }\frac{x^{n}}{\Gamma \left(\kappa n+\mu \right)}.$$

The fractional Maxwell model (4) is uniquely defined by four parameters $\left(E,\tau_{r},\alpha ,\beta \right)$, while the classic Maxwell model (1) is defined by only two parameters (E,η), or equivalently $\left(E,\tau_{r}\right)$.

### 2.4. Spectrum of Relaxation

In rheology, it is commonly assumed that the relaxation modulus G(t) has the following integral representation [11,19]:

(7) $$G\left(t\right)=\int_{0}^{\infty }\frac{ℋ\left(\tau \right)}{\tau }e^{-t/\tau }d\tau ,$$

or, equivalently, by [11]

(8) $$G\left(t\right)=\int_{0}^{\infty }\frac{H\left(v\right)}{v}e^{-tv}dv,$$

where ℋ(τ) and H(v) characterize the distributions of relaxation times τ and relaxation frequencies v, respectively. Equations (7) and (8) yield the formal definitions of the relaxation spectra [11,19], which are related by $H\left(v\right)=ℋ\left(\frac{1}{v}\right)$. Although other definitions of the relaxation spectrum are used in the literature, for example, in [4,21,24,25], the definitions introduced by (7) and (8) dominate.

## 3. Results and Discussion

In this section, the relaxation spectra of the fractional Maxwell model (4) are derived based on the known integral representation of two-parameter Mittag-Leffler function. Next, the monotonicity of the spectra was analyzed, with a special emphasis on the existence of the spectra local extrema. The analysis of the relaxation spectra monotonicity can be reduced to the painstaking analysis of the properties and roots of some cubic function (third order polynomial), whence it has been moved to appendices, where the proofs of most results are given. The necessary and sufficient conditions for the existence of the spectra extrema are derived in the form of two algebraic trigonometric inequalities. Asymptotic properties of the spectra are also examined. A simple scheme for the examining of spectra peaks existence and their determination is outlined. Due to the different forms of spectrum description, two complementary cases, when α<1 and α=1, were studied separately. Direct analytical formulas for the local extrema, minima, and maxima are derived. The influence of FMM parameters on the spectra extrema was investigated by combining analytical and numerical approaches. Finally, the applicability of the relaxation spectra of FMM for describing the unimodal spectra was examined; both Gauss-like and Baumgaertel, Schausberger, and Winter spectra were studied.

### 3.1. Relaxation Spectra of the Fractional Maxwell Model

In [1], the following representation of two-parameter Mittag-Leffler function $E_{\kappa ,\mu }\left(z\right)$ (6) was obtained for complex variable z such that |arg(z)|>πκ, 0<κ≤1 and μ<1+κ [1] (Theorem 4.18, Equation (4.7.17)):

(9) $$E_{\kappa ,\mu }\left(z\right)=\int_{0}^{\infty }K\left(\kappa ,\mu ,r,z\right)dr,$$

where the kernel function [1] (Equation (4.7.15)):

(10) $$K\left(\kappa ,\mu ,r,z\right)=\frac{1}{\pi \kappa }r^{\frac{1-\mu }{\kappa }}e^{-r^{\frac{1}{\kappa }}}\frac{r\sin\left[\pi \left(1-\mu \right)\right]-z\sin\left[\pi \left(1-\mu +\kappa \right)\right]}{r^{2}-2rz\cos\left(\pi \kappa \right)+z^{2}}.$$

Based on (9) and (10), the following result is derived in the Appendix A.1.

**Proposition**  **1.** *Let* 0<β<α≤1. *Then the relaxation time spectrum of the fractional Maxwell model (4) is given by:*

(11) $$ℋ\left(\tau \right)=\frac{E}{\pi }{\left(\frac{\tau }{\tau_{r}}\right)}^{-\beta }\frac{\sin\left(\pi \alpha \right){\left(\frac{\tau }{\tau_{r}}\right)}^{\alpha -\beta }+\sin\left(\pi \beta \right)}{{\left(\frac{\tau }{\tau_{r}}\right)}^{2\left(\alpha -\beta \right)}+2{\left(\frac{\tau }{\tau_{r}}\right)}^{\alpha -\beta }\cos\left[\pi \left(\alpha -\beta \right)\right]+1}\;,$$

*or equivalently by*

(12) $$ℋ\left(\tau \right)=\frac{E}{\pi }{\left(\frac{\tau_{r}}{\tau }\right)}^{\alpha }\frac{{\left(\frac{\tau_{r}}{\tau }\right)}^{\alpha -\beta }\sin\left(\pi \beta \right)+\sin\left(\pi \alpha \right)}{{\left(\frac{\tau_{r}}{\tau }\right)}^{2\left(\alpha -\beta \right)}+2{\left(\frac{\tau_{r}}{\tau }\right)}^{\alpha -\beta }\cos\left(\pi \left(\alpha -\beta \right)\right)+1},$$

*while the spectrum of relaxation frequencies is as follows*

(13) $$H\left(v\right)=\frac{E}{\pi }{\left(\tau_{r}v\right)}^{\alpha }\frac{{\left(\tau_{r}v\right)}^{\alpha -\beta }\sin\left(\pi \beta \right)+\sin\left(\pi \alpha \right)}{{\left(\tau_{r}v\right)}^{2\left(\alpha -\beta \right)}+2{\left(\tau_{r}v\right)}^{\alpha -\beta }\cos\left[\pi \left(\alpha -\beta \right)\right]+1}\;.$$

The last formula can also be obtained by anti-transforming of the Laplace transform of G(t) (5) by using the complex Bromwich formula as outlined, for example, by Mainardi [4,25] for one parameter Mittag-Leffler function.

**Figure 2:**
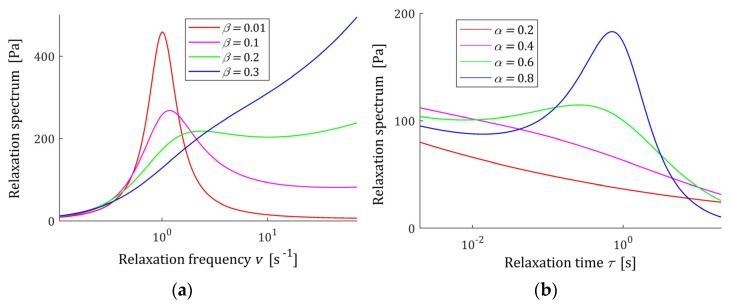
The spectra of the fractional Maxwell model (4) for elastic modulus $E=0.5\times 10^{3}\;\left[\mathrm{Pa}\right]$, relaxation time $\tau_{r}=1\;\left[s\right]$: (**a**) relaxation frequency spectrum H(v) (13) for α=0.9 and (**b**) relaxation time spectrum ℋ(τ) (11) for β=0.1 and the other parameters α, β shown in the plots.

Since undertaken assumptions sin (πβ) and sin (πα) are nonnegative and the expressions from the denominators of (11) and (13) can be expressed in a common compact form

$$x^{2\delta }+2x^{\delta }cos\;\left[\pi \delta \right]+1={\left[x^{\delta }+\cos\;\left(\pi \delta \right)\right]}^{2}+\sin^{2}\;\left(\pi \delta \right),$$

where δ=α−β, the spectra ℋ(τ) and H(v) are nonnegative definite, regardless of the sign of cos [π(α−β)]. A few exemplary relaxation spectra H(v) (13) and ℋ(τ) (11) are shown in Figure 2 for different parameters α and β; the logarithmic scale is applied for the frequencies and times axis. It is seen that the type of their monotonicity depends on parameters α and β, thus on the orders of the stress and strain derivatives in FMM (4). Below, a detailed analysis of the spectra monotonicity is performed, starting with the boundary conditions at v=0 and τ=0 and their asymptotic properties.

Previously, an analytical formula for the relaxation spectrum was obtained for fractional Maxwell model with identical orders of the stress and strain derivatives by Mainardi [4,25] using the complex Bromwich formula to invert the Laplace transform of (5) and bending the Bromwich path into the Hankel path. However, this formula, the properties of which were examined in [41], was derived for another definition of the relaxation spectrum.

### 3.2. Relaxation Spectra of Elementary Fractional Scott-Blair Model

From (3) and (8), by the Laplace transform pair [1] (p. 311)

$$\frac{t^{\mu -1}}{\Gamma \left(\mu \right)}÷\frac{1}{s^{\mu }},\;\mu >0$$

the relaxation frequency spectrum of (2) is obtained

(14) $$H\left(v\right)=\frac{E{\left(\tau_{r}v\right)}^{\alpha }}{\Gamma \left(1-\alpha \right)\Gamma \left(\alpha \right)},$$

whence the relaxation time spectrum is as follows

(15) $$ℋ\left(\tau \right)=\frac{E}{\Gamma \left(1-\alpha \right)\Gamma \left(\alpha \right)}{\left(\frac{\tau_{r}}{\tau }\right)}^{\alpha }.$$

Fixing α and sending β to α, by Equation (13), we obtain

$$H\left(v\right)\to \frac{E}{2\pi }{\left(\tau_{r}v\right)}^{\alpha }\sin\left(\pi \alpha \right)\;,$$

as β→α, whereas, by the reflection equation [1] (Equation (A.1.13))

$$\Gamma \left(1-\alpha \right)\Gamma \left(\alpha \right)=\frac{\pi }{\sin\left(\pi \alpha \right)}\;,$$

the formula follows

(16) $$H\left(v\right)\to \frac{E{\left(\tau_{r}v\right)}^{\alpha }}{2\;\Gamma \left(1-\alpha \right)\Gamma \left(\alpha \right)}$$

Simultaneously, as β→α, Equation (2) results in

$$2\sigma \left(t\right)=E\tau_{r}^{\alpha }\frac{d^{\alpha }\varepsilon \left(t\right)}{dt^{\alpha }},$$

the relaxation spectrum of which, in view of (14), is described by the right-hand side expression of (16). The power nature of the relaxation modulus (3) and the relaxation spectra (15) and (14) characterize the viscoelasticity of many materials; examples are given in [14]. Combined power models may be necessary for complex polymers. Saphiannikova et al. [17] proposed a versatile multi-scale theoretical approach for modelling viscoelasticity of the homogenous rubbers, taking into account relaxation processes at different relaxation time intervals. A four-interval power model with fractional exponents was designated in [17] for a solution-polymerized styrene butadiene rubber.

### 3.3. Monotonicity of the Relaxation Spectra

The boundary conditions are characterized by the next proposition derived in Appendix A.2.

**Proposition**  **2.** *Let* 0<β<α≤1. *Then the relaxation frequency spectrum (13) of the fractional Maxwell model (4) is such that*

(17) H(0)=0,

(18) $$\underset{v\to \infty }{\lim}H\left(v\right)=+\infty \;,$$

*while for the relaxation time spectrum (11) we have*

(19) $$\underset{\tau \to 0^{+}}{\lim}ℋ\left(\tau \right)=+\infty \;,$$

(20) $$\underset{v\to \infty }{\lim}ℋ\left(\tau \right)=0\;.$$

**Figure 3:**
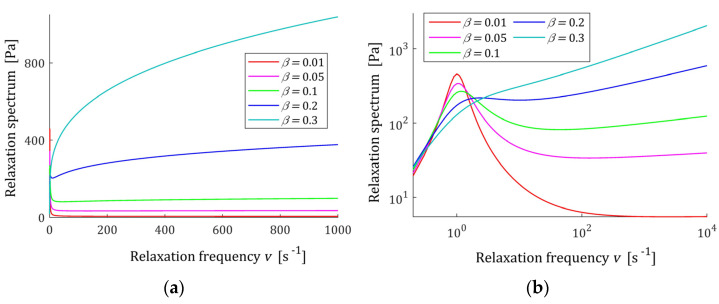
Relaxation frequency spectrum H(v) (13) of the fractional Maxwell model (4) for α=0.9, β=0.01, 0.05, 0.1, 0.2, 0.3, elastic modulus $E=0.5\times 10^{3}\;\left[\mathrm{Pa}\right]$, relaxation time $\tau_{r}=1\;\left(s\right),$ and frequency range $0\leq v\leq v_{m}$, where (**a**) $v_{m}=10^{3}\;\left(s^{-1}\right)$ and (**b**) $v_{m}=10^{4}\;(s^{-1})$.

Both spectra are unbounded. Relaxation frequency spectrum tends, with increasing frequency v, to infinity; however, in view of (A4), the exponent of the power of frequency is equal to max{2β−α,β}, i.e., is smaller than one. A few characteristics H(v) (13) are shown in Figure 3 for two different relaxation frequency range, fixed α and five values of β. However, in view of (18), from a mathematical point of view, spectrum H(v) tends to infinity with growing v, and for physically sensible values of the relaxation frequency, the characteristic H(v) takes a finite value of the order of E.

Relaxation time spectrum is unbounded in the near neighborhood of zero and, in view of (11), with increasing relaxation time τ decays to zero with a negative power law $\tau^{-\beta }$.

The monotonicity properties of both spectra are given below. Models (11) and (13) are described in terms of the following coefficients defined by the model parameters:

(21) $$c_{1}=\sin\left(\pi \beta \right),$$

(22) $$c_{2}=\sin\left(\pi \alpha \right),$$

(23) $$c_{3}=\cos\left[\pi \left(\alpha -\beta \right)\right].$$

Under the assumption 0<β<α≤1, the two first parameters are such that $0<c_{1}\leq 1$ and $0\leq c_{2}\leq 1$, while the sign of $c_{3}$ depends on specific values of α and β. The following coefficients are also defined

(24) $$c_{4}=\left(2\beta -\alpha \right)c_{2}+2\alpha c_{1}c_{3},$$

(25) $$c_{5}=\left(2\alpha -\beta \right)c_{1}+2\beta c_{2}c_{3},$$

to simplify further notations. Using standard trigonometric identities, coefficients $c_{4}$ and $c_{5}$ are expressed as explicit functions of α and β according to:

(26) $$c_{4}=2\beta \sin\left(\pi \alpha \right)+\alpha \sin\left[\pi \left(2\beta -\alpha \right)\right],$$

(27) $$c_{5}=2\alpha \sin\left(\pi \beta \right)+\beta \sin\left[\pi \left(2\alpha -\beta \right)\right].$$

Thus, under taken assumption the coefficient $c_{5}>0$, but the sign of coefficient $c_{4}$ depends on the relationship between the parameters α and β.

The following property, fundamental for the analysis of the spectra monotonicity, results from the comparison of (12) and (13). For a mathematical justification, see Appendix A.3.

**Property** **1.** *Let* 0<β<α≤1. *The relaxation frequency spectrum* H(v) *(13) has a local maximum for relaxation frequency* $v=v_{max}>0$ *and a local minimum for frequency* $v=v_{min}>0$*, if and only if the relaxation time spectrum* ℋ(τ) *(11) has a local maximum for the time* $\tau =\tau_{max}=\frac{1}{v_{max}}>0$ *and a local minimum for* $\tau =\tau_{min}=\frac{1}{v_{min}}>0$*. Spectrum* H(v) *is a monotonically increasing function if and only if spectrum* ℋ(τ) *monotonically decreases*.

Thus, the monotonicity of spectrum H(v) uniquely determines the monotonicity of spectrum ℋ(τ), and vice versa. The first simple, useful, necessary but not sufficient condition for the existence of local extrema of H(v) and ℋ(τ) is proved in the Appendix A.4.

**Proposition**  **3.** *Let* 0<β<α≤1. *If the relaxation time* ℋ(τ) *(11) and frequency* H(v) *(13) spectra of the fractional Maxwell model (4) have local extrema for some times* τ>0 *and frequencies* v>0*, then the coefficient* $c_{4}<0$*, i.e., the following inequality holds*

(28) 2βsin(πα)<αsin[π(α−2β)].

Thus, if inequality (28) is not satisfied, then by simple contradiction, relaxation spectra H(v) (13) and ℋ(τ) (11) are, respectively, monotonically increasing and decreasing functions. Inequality (28) implies the next, weaker, necessary condition of the existence of the spectra local extrema, namely, the requirement that β<α/2.

It is demonstrated in Appendix A.5 that the further analysis, concerning the existence of the spectra extrema is convenient to carry out separately in two different cases when α is equal to one, or not. The analysis begins with the second case.

### 3.4. Analysis of the Relaxation Spectra Monotonicity for α<1

Bearing in mind Proposition 3, assume for further analysis that $c_{4}<0$. The existence of the spectra local extrema is uniquely resolved by the following necessary and sufficient condition proved in Appendix A.6.

**Proposition**  **4.** *Let* 0<β<α<1 be such that $c_{4}<0$. *The relaxation time* ℋ(τ) *(11) and frequency* H(v) *(13) spectra of the fractional Maxwell model (4) have local minima and maxima for positive arguments if and only if*

(29) $${\left[\frac{c_{4}^{3}}{27{\left[\beta c_{1}\right]}^{3}}-\frac{c_{4}c_{5}}{6{\left[\beta c_{1}\right]}^{2}}+\frac{\alpha c_{2}}{2\beta c_{1}}\right]}^{2}+{\left[\frac{3\beta c_{1}c_{5}-c_{4}^{2}}{9{\left[\beta c_{1}\right]}^{2}}\right]}^{3}<0,$$

*where the coefficients* $c_{1}$, $c_{2}$, $c_{4,}$ *and* $c_{5}$ *are defined by (21), (22), (24), and (25), respectively. In the opposite case, when the inequality*

(30) $${\left[\frac{c_{4}^{3}}{27{\left[\beta c_{1}\right]}^{3}}-\frac{c_{4}c_{5}}{6{\left[\beta c_{1}\right]}^{2}}+\frac{\alpha c_{2}}{2\beta c_{1}}\right]}^{2}+{\left[\frac{3\beta c_{1}c_{5}-c_{4}^{2}}{9{\left[\beta c_{1}\right]}^{2}}\right]}^{3}\geq 0,$$

*holds, then the relaxation frequency spectrum* H(v) *(13) is monotonically increasing function, while the relaxation time spectrum* ℋ(τ) *(11) is monotonically decreasing*.

Since for 0<β<1 the denominators in all fractions of the right-hand side of inequality (29) are positive, this inequality can be rewritten in a more useful way for numerical verification in an equivalent form

(31) Ξ(α,β)<0,

where

(32) $$\Xi (\alpha ,\beta )={\left[2c_{4}^{3}-9\beta c_{1}c_{4}c_{5}+27\alpha c_{2}{\left(\beta c_{1}\right)}^{2}\right]}^{2}+4{\left[3\beta c_{1}c_{5}-c_{4}^{2}\right]}^{3}.$$

From the above proposition, in particular from inequality (29), the next necessary condition for existence of the spectra local extrema follows; for derivation see Appendix A.7.

**Proposition**  **5.** *Let* 0<β<α<1 *be such that* $c_{4}<0$. *If the relaxation frequency* H(v) *(13) and time* ℋ(τ) *(11) spectra of the fractional Maxwell model (4) have local extrema for some frequencies* v>0 *and times* τ>0*, then the following inequality holds*

(33) $$3\beta c_{1}c_{5}<c_{4}^{2},$$

*where the coefficients* $c_{1}$*,* $c_{4}$*, and* $c_{5}$ *are defined by (21), (24), and (25), which can be expressed in equivalent form*

(34) $$\begin{array}{c} \beta^{2}\cos(2\pi \alpha )-2\beta \alpha \cos(2\pi \beta )-\beta (4-3\beta )\cos[2\pi (\alpha -\beta )]+\\ \alpha^{2}\cos[2\pi (2\beta -\alpha )]<\alpha^{2}+4\beta^{2}-6\beta \alpha . \end{array}$$

From Propositions 3, 4, and 5, the following simple scheme was followed to check if there were local extrema of the relaxation spectra for given parameters α and β.

1. Check if the inequality $c_{4}<0$, or equivalent (28), holds. If yes, go to step 2. Otherwise, go to step 4.
2. Check if the inequality (33), or equivalent (34), holds. If yes, go to step 3. Otherwise, go to step 4.
3. Check if the inequalities $c_{4}<0$ and (29), or equivalent (31), hold. If yes, a local extrema of both spectra H(v) and ℋ(τ) exists. Otherwise, go to step 4.
4. Spectrum H(v) is a monotonically increasing function for all v>0, while spectrum ℋ(τ) is a monotonically decreasing function for all τ>0.

Checking in steps 1 and 2, if $c_{4}<0$, equivalently (28), and next (33), hold, avoids verification of the necessary and sufficient condition (29) in the case when they are not satisfied.

**Figure 4:**
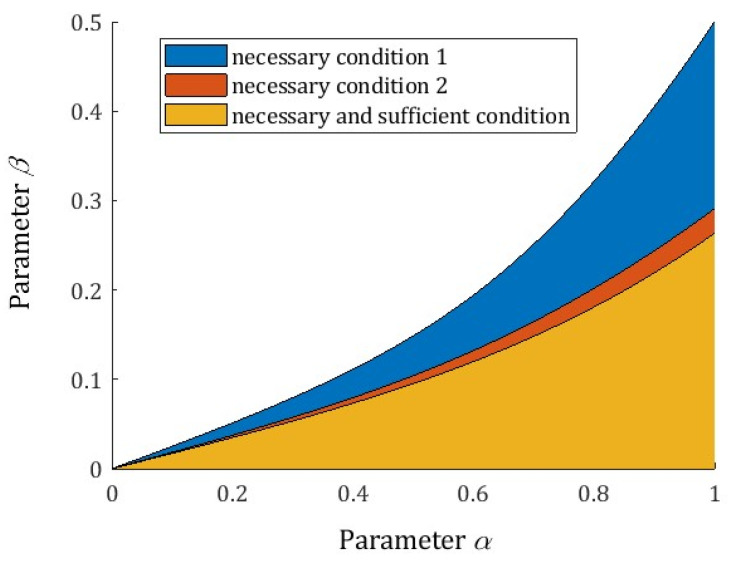
The sets of the derivative orders parameters α and β fulfilling the necessary and sufficient conditions for the existence of the local extrema of the relaxation spectra H(v) (13) and ℋ(τ) (11) of the fractional Maxwell model (4): necessary condition 1—$c_{4}<0$ (equivalently (28)), necessary condition 2—(33) and necessary and sufficient conditions $c_{4}<0$ and (29).

Both the necessary and sufficient conditions are formulated in terms of the α and β parameters; they do not depend on the rheological model parameters *E* and $\tau_{r}$. The sets of the derivative order parameters α and β for which the necessary conditions (28) and (33) hold are depicted in Figure 4, together with the set of all parameters α and β, for which the local extrema of the spectra exist. As can be seen, the necessary and sufficient condition (33) of Proposition 5 is a good approximation of the necessary and sufficient conditions of the extrema existence specified by Proposition 4.

Below, the spectra extrema are determined and examined, separately, for α<1 and α=1.

### 3.5. Extrema of the Relaxation Spectra for α<1

The following property results directly from Property 1 and the proofs of Propositions 3 and 4.

**Proposition**  **6.** *Let the parameters* α *and* β *be such that inequalities* 0<β<α<1, $c_{4}<0$ *and (29) are satisfied. Then:*

***(i)***  *The relaxation frequency spectrum* H(v) *(13) of the fractional Maxwell model (4) has the local maximum*
  (35) $$v_{max}=\frac{1}{\tau_{r}}{\left(x_{3}\right)}^{\frac{1}{\alpha -\beta }},$$
  *and the local minimum* $v_{min}>v_{max}$ *given by*
  (36) $$v_{min}=\frac{1}{\tau_{r}}{\left(x_{2}\right)}^{\frac{1}{\alpha -\beta }},$$
  *when the inequality holds*
  (37) $$\frac{c_{4}^{3}}{27{\left[\beta c_{1}\right]}^{3}}-\frac{c_{4}c_{5}}{6{\left[\beta c_{1}\right]}^{2}}+\frac{\alpha c_{2}}{2\beta c_{1}}\geq 0,$$
  *and equal to*
  (38) $$v_{min}=\frac{1}{\tau_{r}}{\left(x_{1}\right)}^{\frac{1}{\alpha -\beta }},$$
  *in the case opposite to inequality (37), where*
  (39) $$x_{1}=-2r\cos\left(\frac{\theta }{3}\right)-\frac{c_{4}}{3\beta c_{1}},$$
  (40) $$x_{2}=2r\cos\left(\frac{\pi -\theta }{3}\right)-\frac{c_{4}}{3\beta c_{1}},$$
  (41) $$x_{3}=2r\cos\left(\frac{\pi +\theta }{3}\right)-\frac{c_{4}}{3\beta c_{1}},$$
  *with*
  (42) $$r=sgn\left(\frac{c_{4}^{3}}{27{\left[\beta c_{1}\right]}^{3}}-\frac{c_{4}c_{5}}{6{\left[\beta c_{1}\right]}^{2}}+\frac{\alpha c_{2}}{2\beta c_{1}}\right)\sqrt{\left|\frac{3\beta c_{1}c_{5}-c_{4}^{2}}{9{\left[\beta c_{1}\right]}^{2}}\right|},$$
  *and the angle* θ *defined by*
  (43) $$cos\left(\theta \right)=\frac{\frac{c_{4}^{3}}{27{\left[\beta c_{1}\right]}^{3}}-\frac{c_{4}c_{5}}{6{\left[\beta c_{1}\right]}^{2}}+\frac{\alpha c_{2}}{2\beta c_{1}}}{r^{3}},$$
  *where the coefficients* $c_{1}$, $c_{2}$, $c_{4}$*, and* $c_{5}$ *are defined by (21), (22), (24), and (25), respectively,* sgn(·)*. denotes signum function.*
***(ii)***  *The relaxation time spectrum* ℋ(τ) *(11) has the local maximum*
  (44) $$\;\tau_{max}=\frac{\tau_{r}}{{\left(x_{3}\right)}^{\frac{1}{\alpha -\beta }}},$$
  *and the local minimum* $\tau_{min}<\tau_{max}$ *given by*
  (45) $$\tau_{min}=\frac{\tau_{r}}{{\left(x_{2}\right)}^{\frac{1}{\alpha -\beta }}},$$
  *when the inequality (37) holds, while in the opposite case equal to*
  (46) $$\tau_{min}=\frac{\tau_{r}}{{\left(x_{1}\right)}^{\frac{1}{\alpha -\beta }}}.$$

**Figure 5:**
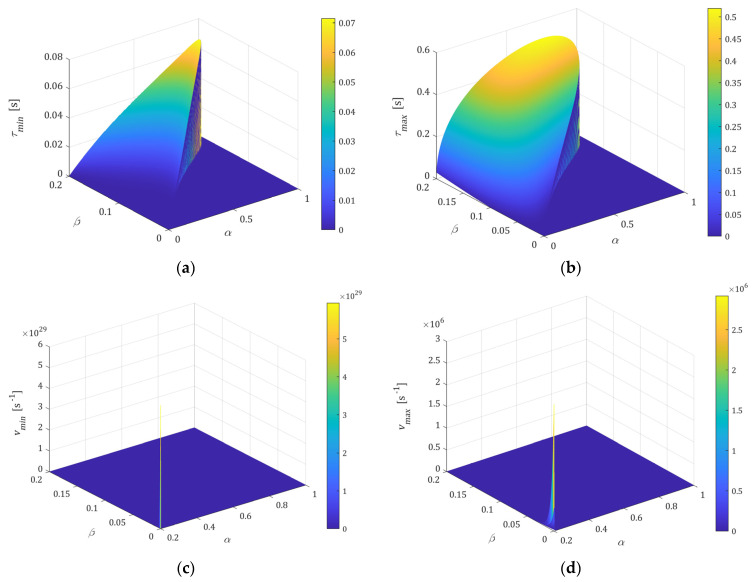
The local extrema of the relaxation spectra: (**a**) minimum $\tau_{min}$ (45), (46), and (**b**) maximum $\tau_{max}$ (44) of relaxation time spectrum ℋ(τ) (11) for parameters 0<β<α<1; (**c**) minimum $v_{min}$ (36), (38), and (**d**) maximum $v_{max}$ (35) of the relaxation frequency spectrum H(v) (13) for parameters 0.2<α<1. Fixed relaxation time $\tau_{r}=1\;\left[s\right]$. For α and β, such that spectrum H(v) monotonically increases, the plot is equal to zero.

**Figure 6:**
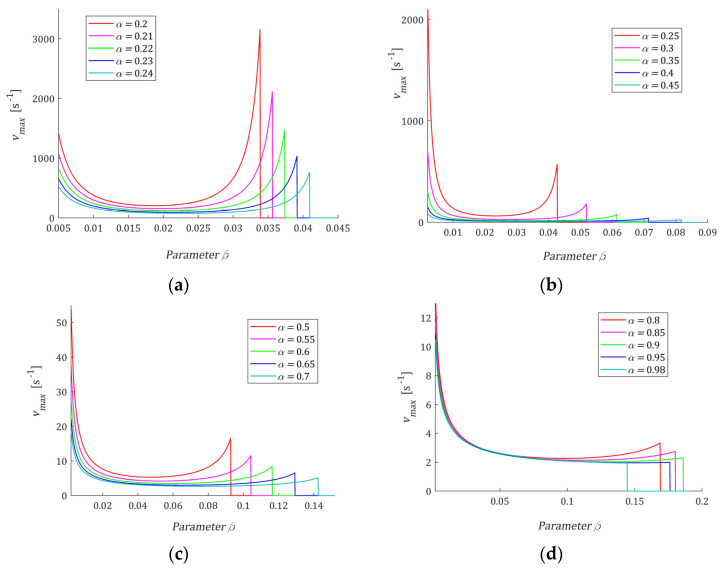
The local maximum $v_{max}$ (35) of the relaxation frequency spectrum H(v) (13) as a function of parameter β for for: (**a**) *α* = 0.2, 0.21, 0.22, 0.23, 0.24; (**b**) *α* = 0.25, 0.3, 0.35, 0.4, 0.45; (**c**) *α* = 0.5, 0.55, 0.6, 0.65, 0.7; (**d**) *α* = 0.8, 0.85, 0.9, 0.95, 0.98; for *α* and *β*, such that spectrum *H(v)* monotonically increases, the plot is equal to zero. Relaxation time $\tau_{r}=1\;\left[s\right]$.

The relaxation time $\tau_{r}$ affects the E-independent extrema $\tau_{min}$, $\tau_{max}$, $v_{min}$, and $v_{max}$. Dependence of the extrema on α and β is illustrated by the following figures. Figure 5a,b shows the local minimum $\tau_{min}$ (45), (46), and maximum $\tau_{max}$ (44) for 0< *β* < α < 1; for α and β such that spectrum ℋ(τ) monotonically decreases, the plot is equal to zero. The colors are specified by color bar added to the right. Figure 5c,d illustrate for 0.2<α<1 the range of variation of $v_{max}$ (35) and $v_{min}$ (36), (38) as functions of α and β varying from the values close to zero to that on the order of 106 and 1029, respectively. Dependence of $v_{max}$ (35) and $v_{min}$ (36), (38) on parameter 0<β<α for a few α is depicted, separately, in Figure 6. However, from a practical point of view, mainly $v_{max}$ is important, and this varies within the frequencies for which the real spectra peaks occur. The selection of parameters α and β, and even only β for a given α, allows us to shape the spectrum whose maximum peak varies in a very wide range of frequencies.

**Figure 7:**
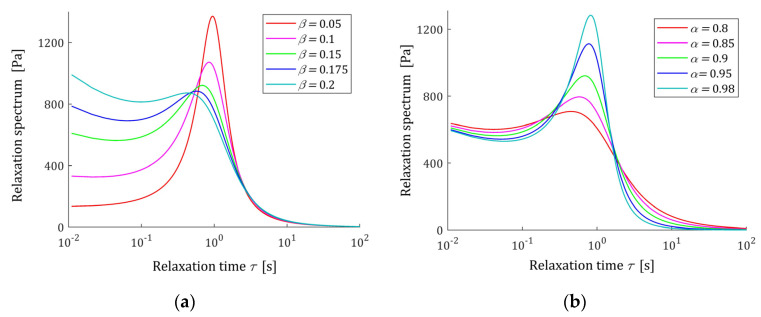
Relaxation time spectrum ℋ(τ) (11) of the fractional Maxwell model (4) for: (**a**) α=0.9 and β=0.05, 0.1, 0.15, 0.2, 0.25; (**b**) β=0.15 and α=0.8, 0.85, 0.9, 0.95, 0.98. Elastic modulus $E=0.5\times 10^{4}\;\left[\mathrm{Pa}\right]$, and relaxation time $\tau_{r}=1\;\left[s\right]$.

**Figure 8:**
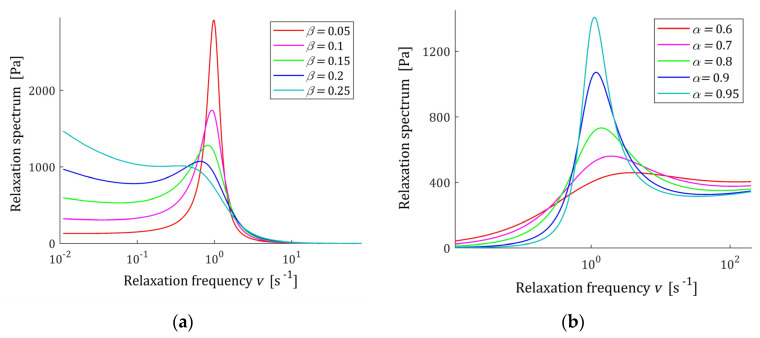
Relaxation frequency spectrum H(v) (13) of the fractional Maxwell model (4) for: (**a**) α=0.98 and β=0.05, 0.1, 0.15, 0.2, 0.25; (**b**) *β* = 0.11 and α=0.6, 0.7, 0.8, 0.9, 0.95. Elastic modulus $E=0.5\times 10^{4}\;\left[\mathrm{Pa}\right]$, and relaxation time $\tau_{r}=1\;\left[s\right]$.

The course of the spectrum ℋ(τ) (11) is illustrated by Figure 7. In Figure 7a, the spectrum ℋ(τ) is depicted for a few values of β for fixed parameter α, while in Figure 7b parameter β is fixed and the spectrum’s ℋ(τ) dependence on changing α is illustrated. In Figure 8 the relaxation frequency spectra H(v) (13) are given for other values of fixed parameters α and β. The non-integer orders α and β uniquely determine the existence or absence of local extrema of the relaxation spectra of the FMM model and, together with the relaxation time $\tau_{r}$, the values of local minima and maxima. The smaller the β is the higher their local maxima, and the more concise their peaks are. Conversely, the greater the α, the higher the maxima and the more pointed peaks.

### 3.6. Analysis of the Relaxation Spectra Monotonicity for α=1

For α=1, the relaxation spectrum ℋ(τ) (11) is given by

(47) $$ℋ\left(\tau \right)=\frac{E}{\pi }{\left(\frac{\tau }{\tau_{r}}\right)}^{-\beta }\frac{\sin\left(\pi \beta \right)}{{\left(\frac{\tau }{\tau_{r}}\right)}^{2\left(1-\beta \right)}-2{\left(\frac{\tau }{\tau_{r}}\right)}^{1-\beta }\cos\left(\pi \beta \right)+1}\;,$$

or according to (12) by the formula

(48) $$ℋ\left(\tau \right)=\frac{E}{\pi }\frac{{\left(\frac{\tau_{r}}{\tau }\right)}^{2-\beta }\sin\left(\pi \beta \right)}{{\left(\frac{\tau_{r}}{\tau }\right)}^{2\left(1-\beta \right)}-2{\left(\frac{\tau_{r}}{\tau }\right)}^{1-\beta }\cos\left(\pi \beta \right)+1},$$

while, by (13), spectrum H(v) is described by

(49) $$H\left(v\right)=\frac{E}{\pi }\frac{{\left(\tau_{r}v\right)}^{2-\beta }\sin\left(\pi \beta \right)}{{\left(\tau_{r}v\right)}^{2\left(1-\beta \right)}-2{\left(\tau_{r}v\right)}^{1-\beta }\cos\left(\pi \beta \right)+1}\;.$$

For α=1, by (26), the necessary condition for the existence of the extrema specified in Proposition 3, i.e., $c_{4}<0$, is equivalent to

$$c_{4}=\sin\left[\pi \left(2\beta -1\right)\right]=-\sin\left(2\beta \pi \right)<0,$$

i.e., is fulfilled whenever $\beta <\frac{1}{2}$.

The monotonicity of the spectra is resolved by the next result proved in Appendix A.8. The necessary and sufficient conditions for the existence of local extrema and formulas describing them are given.

**Proposition**  **7.** *Let* $0<\beta <\frac{1}{2}$ *and *α* = 1. The spectra of relaxation frequencies* H(v) *(49) and times* ℋ(τ) *(47) of the fractional Maxwell model (4) have local minima and maxima for positive arguments, if and only if parameter* β *is such that the following inequality holds*

(50) $$\cos^{2}\left(\pi \beta \right)>\beta \left(2-\beta \right).$$

*Then:*

***(i)***  *The relaxation frequency spectrum* H(v) *(49) has the local maximum*
  (51) $$v_{max}=\frac{1}{\tau_{r}}{\left[\frac{\cos\left(\pi \beta \right)-\sqrt{\cos^{2}\left(\pi \beta \right)-\beta \left(2-\beta \right)}}{\beta }\right]}^{\frac{1}{1-\beta }},$$
  *and the local minimum* $v_{min}>v_{max}$ *given by*
  (52) $$v_{min}=\frac{1}{\tau_{r}}{\left[\frac{\cos\left(\pi \beta \right)+\sqrt{\cos^{2}\left(\pi \beta \right)-\beta \left(2-\beta \right)}}{\beta }\right]}^{\frac{1}{1-\beta }}.$$
***(ii)***  *The relaxation time spectrum* ℋ(τ) *(47) has the local maximum*
  (53) $$\;\tau_{max}=\frac{\tau_{r}}{{\left[\frac{\cos\left(\pi \beta \right)-\sqrt{\cos^{2}\left(\pi \beta \right)-\beta \left(2-\beta \right)}}{\beta }\right]}^{\frac{1}{1-\beta }}},$$
  *and the local minimum* $\tau_{min}<\tau_{max}$ *given by*
  (54) $$\tau_{min}=\frac{\tau_{r}}{{\left[\frac{\cos\left(\pi \beta \right)+\sqrt{\cos^{2}\left(\pi \beta \right)-\beta \left(2-\beta \right)}}{\beta }\right]}^{\frac{1}{1-\beta }}}.$$
  *If* β *is such that inequality (50) does not hold, then* ℋ(τ) *(47) and* H(v) *(49) are monotonically decreasing and increasing functions, respectively*.

A complete set of $0<\beta <\frac{1}{2}$ for which the necessary and sufficient condition (50) holds is as follows: 0<β<0.263516.

### 3.7. Extrema of the Relaxation Spectra for α=1

**Figure 9:**
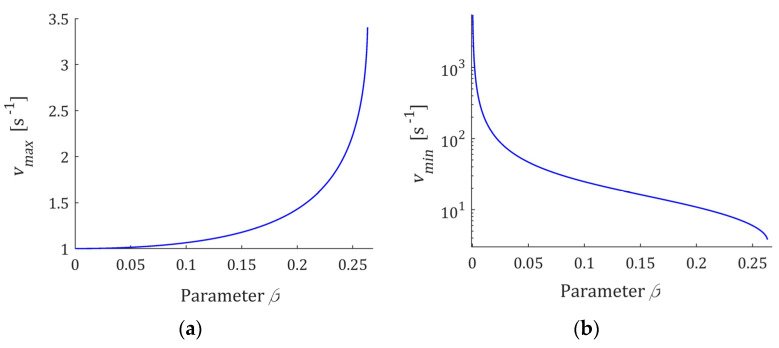

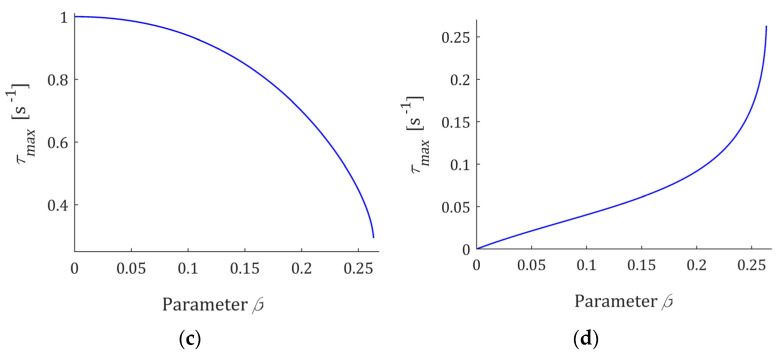
The local extrema: (**a**) $v_{max}$ (51); (**b**) $v_{min}$ (52); (**c**) $\tau_{max}$ (53); (**d**) $\tau_{min}$ (54) of the relaxation frequency H(v) (49) and time ℋ(τ) (47) spectra as the functions of parameter β fulfilling the necessary and sufficient condition (50). Relaxation time $\tau_{r}=1\;\left[s\right]$.

The frequencies $v_{max}$, $v_{min}$ and the times $\tau_{max}$, $\tau_{min}$ are uniquely determined by β and $\tau_{r}$. The extrema as functions of the parameter β are shown in Figure 9 for β satisfying the necessary and sufficient condition (50); the relaxation time $\tau_{r}=1\;\left[s\right]$ is assumed. In Figure 9b, for $v_{min}$, the logarithmic scale is applied. The peak frequency $v_{max}$ increases with increasing frequency, therefore $\tau_{max}$ decreases. Decreasing with increasing β, $v_{min}$ means that spectrum H(v) (49) increases monotonically to infinity for lower relaxation frequencies. In turn, being smaller with increasing β times $\tau_{min}$ means that spectrum ℋ(τ) (47) decreases faster for relaxation times smaller than $\tau_{min}$.

Since, in view of (51) and (53), $v_{max}=1/\tau_{max}$, by (49) and (48), the equality $H\left(v_{max}\right)=ℋ\left(\tau_{max}\right)$ holds. Similarly, $H\left(v_{min}\right)=ℋ\left(\tau_{min}\right)$. By (51) and (49), the local maximum of the spectra is as follows

(55) $$H\left(v_{max}\right)=ℋ\left(\tau_{max}\right)=\frac{E}{\pi \left(1-\beta \right)\beta^{\frac{\beta }{1-\beta }}}\frac{{\left[\cos\left(\pi \beta \right)-\sqrt{\cos^{2}\left(\pi \beta \right)-\beta \left(2-\beta \right)}\right]}^{\frac{2-\beta }{1-\beta }}\sin\left(\pi \beta \right)}{\cos^{2}\left(\pi \beta \right)-\beta -\cos\left(\pi \beta \right)\sqrt{\cos^{2}\left(\pi \beta \right)-\beta \left(2-\beta \right)}},$$

while, in view of (52), local minimum is given by

(56) $$H\left(v_{min}\right)=ℋ\left(\tau_{min}\right)=\frac{E}{\pi \left(1-\beta \right)\beta^{\frac{\beta }{1-\beta }}}\frac{{\left[\cos\left(\pi \beta \right)+\sqrt{\cos^{2}\left(\pi \beta \right)-\beta \left(2-\beta \right)}\right]}^{\frac{2-\beta }{1-\beta }}\sin\left(\pi \beta \right)}{\cos^{2}\left(\pi \beta \right)-\beta +\cos\left(\pi \beta \right)\sqrt{\cos^{2}\left(\pi \beta \right)-\beta \left(2-\beta \right)}}\;.$$

**Figure 10:**
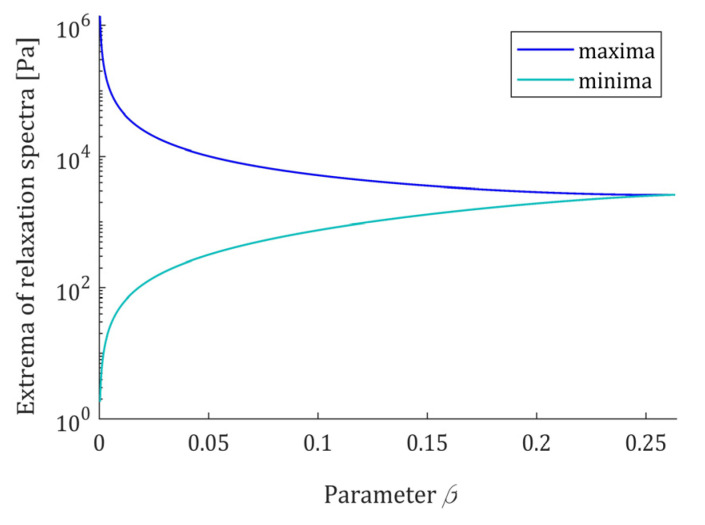
The local maxima $H\left(v_{max}\right)=ℋ\left(\tau_{max}\right)$ (55) and minima $H\left(v_{min}\right)=ℋ\left(\tau_{min}\right)$ (56) of the relaxation spectra H(v) (49) and ℋ(τ) (47), as the functions of parameter β fulfilling the necessary and sufficient condition (50). Elastic modulus $E=0.5\times 10^{4}\;\left[\mathrm{Pa}\right]$.

Thus, for 0<β<0.263516 the quotient

$$\frac{H\left(v_{max}\right)}{H\left(v_{min}\right)}=\frac{\cos^{2}\left(\pi \beta \right)-\beta +\cos\left(\pi \beta \right)\sqrt{\cos^{2}\left(\pi \beta \right)-\beta \left(2-\beta \right)}}{\cos^{2}\left(\pi \beta \right)-\beta -\cos\left(\pi \beta \right)\sqrt{\cos^{2}\left(\pi \beta \right)-\beta \left(2-\beta \right)}}\cdot \frac{{\left[\cos\left(\pi \beta \right)-\sqrt{\cos^{2}\left(\pi \beta \right)-\beta \left(2-\beta \right)}\right]}^{\frac{2-\beta }{1-\beta }}}{{\left[\cos\left(\pi \beta \right)+\sqrt{\cos^{2}\left(\pi \beta \right)-\beta \left(2-\beta \right)}\right]}^{\frac{2-\beta }{1-\beta }}},$$

monotonically decreases, from infinity to one. The maxima (55) and minima (56) are uniquely determined by β and elastic modulus E. Since they are proportional to E, only the dependence on parameter β is illustrated in Figure 10 for fixed E; a logarithmic scale was used for the vertical axis.

**Figure 11:**
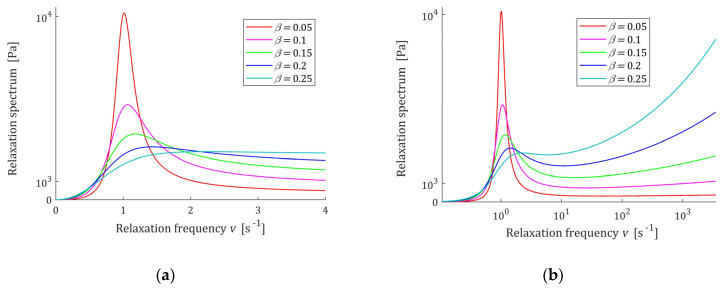
Relaxation frequency spectrum H(v) (49) of the of the fractional Maxwell model (4) for α=1, β=0.05, 0.1, 0.15, 0.2, 0.25, elastic modulus $E=0.5\times 10^{4}\;\left(\mathrm{Pa}\right)$, $\tau_{r}=1\;\left(s\right)$, and frequency range $0\leq v\leq v_{m}$, where: (**a**) $v_{m}=4\;\left(s^{-1}\right)$ and (**b**) $v_{m}=3.5\times 10^{3}\;\left(s^{-1}\right)$.

**Figure 12:**
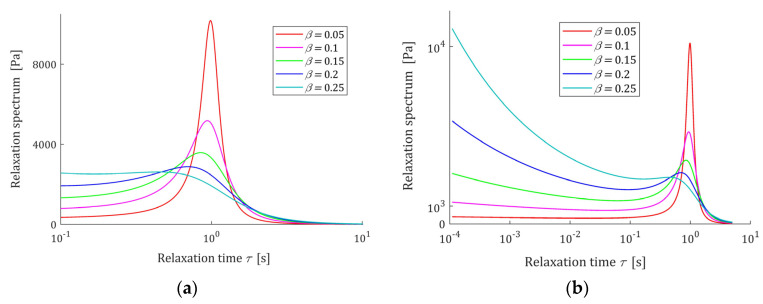
Relaxation time spectrum ℋ(τ) (47) of the fractional Maxwell model (4) for α=1, β=0.05, 0.1, 0.15, 0.2, 0.25, $E=0.5\times 10^{4}\;\left(\mathrm{Pa}\right)$, $\tau_{r}=1\;\left(s\right)$, and range of times: (**a**) $10^{-1}<\tau \leq 10$ and (**b**) $10^{-4}<\tau \leq 5\;\left(s\right)$.

In conclusion, the relaxation time $\tau_{r}$ and parameter β uniquely determine the extrema relaxation times and frequencies. In turn, the extreme values of the spectra depend on the elastic modulus *E* and β. The course of the spectrum H(v) (49) is illustrated by Figure 11, where the spectrum H(v) (49) is depicted for a few values of β; in Figure 11b the logarithmic scale is used for the relaxation frequency axis to expose both the maxima and minima of the characteristics. In Figure 12, the relaxation time spectra ℋ(τ) are given for the same parameters β; the logarithmic scale is used for the relaxation times axis.

From Figure 11 and Figure 12 it is seen that the lower β is, the higher its local maximum $H\left(v_{max}\right)=ℋ\left(\tau_{max}\right)$ is, and the more concise this peak is. Thus, the order parameter β influences both the ‘height’ of the spectrum peak and its ‘width’. The relaxation frequencies and times of the peaks also depends on the relaxation time $\tau_{r}$—the bigger their ‘height’, the bigger the elastic modulus E is. Therefore, by the respective choice of the three model parameters $\left(E,\;\tau_{r},\beta \;\right)$, a wide class of the unimodal relaxation spectra can be described.

### 3.8. Identification

The spectrum, not being directly accessible by measurement, is recovered from relaxation stress [42,43,44] or oscillatory shear data [19,29,45,46] by using an appropriate identification method. Identification consists of selecting, within the chosen class of models given by (4) with the relaxation modulus described by (5), such a model, which ensures the best approximation to the measurement data. To clarify the description, model G(t) (5) is denoted as

(57) $$G_{M}\left(t,g\right)=E{\left(\frac{t}{\tau_{r}}\right)}^{-\beta }E_{\alpha -\beta ,1-\beta }\left(-{\left(\frac{t}{\tau_{r}}\right)}^{\alpha -\beta }\right),$$

where the subscript ‘M’ means the model and

(58) $$g={\left[\begin{array}{cccc} \alpha & \beta & E & \tau_{r} \end{array}\right]}^{T}$$

is a 4-element vector of unknown coefficients of the model. The relaxation time spectrum ℋ(τ) (11) for parameters g (58) will be hereinafter referred to as ℋ(τ,g), by analogy spectrum of the relaxation frequency H(v) (13) as H(v,g ), to emphasize the dependence on the determined parameters of the model.

Suppose a certain identification experiment (stress relaxation test [11,35,37]) resulted in a set of measurements of the relaxation modulus $\left\{\bar{G}\left(t_{i}\right)=G\left(t_{i}\right)+z\left(t_{i}\right)\right\}$ at the sampling instants $t_{i}\geq 0$, i=1,…,N, where $z\left(t_{i}\right)$ is the measurement noise. Following [44,47], as a measure of the model accuracy, the mean quadratic index is taken

(59) $$Q_{N}\left(g\right)=\frac{1}{N}\sum_{i=1}^{N}{\left[\bar{G}\left(t_{i}\right)-G_{M}\left(t_{i},g\right)\right]}^{2}.$$

Thus, the optimal identification of FMM model defined by (4) or (57) consists of determining the model parameters minimizing the index $Q_{N}\left(g\right)$, i.e., in solving the non-linear least-squares problem

(60) $$\underset{g}{\min}\;Q_{N}\left(g\right)=Q_{N}\left(g^{*}\right).$$

When the optimal parameter $g^{*}$ is determined, the spectra of FMM are described by $ℋ\left(\tau ,g^{*}\right)$ and $H\left(v,g^{*}\right)$ according to the formulas (11) and (13), respectively.

There are known methods of identifying FMM either by direct minimization of the index $Q_{N}\left(g\right)$, or by approximate identification methods, according to which the original identification task (60) is replaced by a simpler task that gives an approximate solution. An example of such a method is the scheme proposed by Stankiewicz [18]. However, the task itself (60) is not the subject of this paper, so it will not be discussed in detail here. Since it was desirable to accurately determine the model of the relaxation modulus $G_{M}\left(t,g\right)$ (57), the function MLFFIT2 provided by Podlubny [48] for fitting data using the two parameters Mittag-Leffler function multiplied by a power function was used to determine it. This procedure has been introduced and described in detail in [49]. All four parameters of the model will be selected optimally.

Both unimodal and multimodal, especially bimodal, relaxation spectra are used to describe viscoelastic properties of polymers. Bearing in mind the unimodal character of the spectra (11) and (13), the applicability of these spectra for describing commonly used models of polymer spectra was examined. Both Gauss-like distributions and BSW spectra dominating in the rheology of polymers [36,50] were considered. All models were simulated in Matlab R2022a, using the special function *erfc* for the Gauss-like distributions. Functions MLFFIT2 [48] and MLF [51], provided by Podlubny, were used.

### 3.9. Applicability of the FMM Spectra to Modelling Gaussian Spectra

In this section, relaxation spectra of the fractional Maxwell model are applied to modelling the relaxation spectra described by the unimodal Gauss-like distributions. Although studies confirming the use of the BSW spectrum for various polymers prevail, thereby research concerning them does not require justification, there are also studies indicating the use of the Gaussian spectrum for some polymeric materials, including biopolymers. In [52], the linear viscoelastic behavior of commercial polypropylenes is studied under the assumption that the relaxation spectrum takes the shape of a log-normal distribution, which is in agreement with the linear viscoelasticity theory by providing limiting values, contrary to BSW model. Museau et al. [32] applied a Gaussian distribution of the relaxation times, modified to introduce asymmetry of the relaxation process and to describe viscoelasticity in poly(methyl methacrylate). Recently, the spectra of a Gaussian character for bimodal polyethylene were determined by Kwakye-Nimo et al. [33] (Figures 4b and 8b), for glass by Wang et al. [53] (Figure 2), and for soft polyacrylamide gels by Pérez-Calixto et al. [35] (Figure A4). The spectra of various biopolymers studied by many researchers are also Gaussian in nature, for example, some (wheat, potato, corn, and banana) native starch gels [34] (Figures 6b, 7 and 9a), xanthan gum water solution [36] (Figures 6 and 10), carboxymethylcellulose (CMC) [36] (Figures 6 and 11), wood [54] (Figure 7), and [55] (Figures 2 and 3), fresh egg white-hydrocolloids [36] (Figures 6 and 14). Gauss-type spectra have been tested when developing new viscoelastic models and identification methods, for example, in [56] (Figure 2), [57] (Figures 9, 11, and 17) and [58] (Figures 2, 3, 6, 7–11, and 14). Two examples with different relaxation times are shown.

#### 3.9.1. Example 1

Consider the viscoelastic material of relaxation spectrum described by the unimodal Gauss-like distribution:

(61) $$ℋ\left(\tau \right)=\vartheta e^{-{\left(\frac{1}{\tau }-m\right)}^{2}/q}/\tau ,$$

where the parameters are as follows [44]: ϑ=31520 Pa·s, $m=0.0912{\;s}^{-1}$ and $q=3.25\times 10^{-3}{\;s}^{-2}$. The related relaxation modulus is [34]:

(62) $$G\left(t\right)=\frac{\sqrt{\pi q}}{2}\vartheta \;e^{\frac{1}{4}t^{2}q-mt}erfc\left(\frac{\frac{1}{2}tq-m}{\sqrt{q}}\right).$$

**Figure 13:**
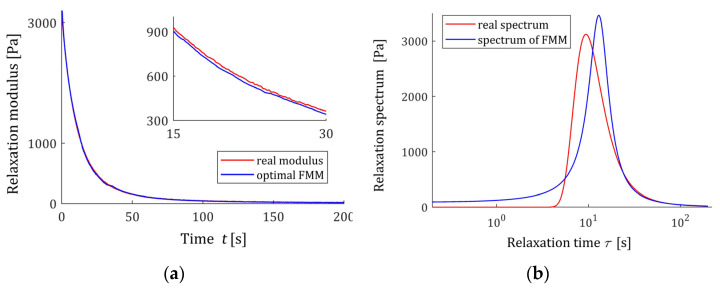
For the “real” material from Example 1 and the fractional Maxwell model (57) with optimal parameters $g^{*}$ (63) are presented: (**a**) the measurements $\bar{G}\left(t_{i}\right)$ of the real relaxation modulus G(t) (62) (red points) and model $G_{M}\left(t,g^{*}\;\right)$ (57); (**b**) real relaxation time spectrum ℋ(τ) (61) (solid red line) and the spectrum model $ℋ\left(\tau ,g^{*}\;\right)$ (11).

In the experiment, N=1000 sampling instants $t_{i}$ were generated with the constant period in the time interval T=[0, 200] with seconds chosen in view of the course of the modulus G(t) (62). Additive measurement noises $z\left(t_{i}\right)$ were selected independently by random choice with uniform distribution on the interval [−5, 5] Pa. The optimal parameters of the model (57) are determined

(63) $$g^{*}={\left[\begin{array}{cccc} \alpha^{*} & \beta^{*} & E^{*} & \tau_{r}^{*} \end{array}\right]}^{T}={\left[\begin{array}{cccc} 0.935 & 0.025 & 3.0682\times 10^{3}\;\mathrm{Pa} & 13.13358\;s \end{array}\right]}^{T},$$

the mean square relative identification index defined by

(64) $$J_{N}\left(g\right)=\frac{1}{N}\sum_{i=1}^{N}\frac{{\left[\bar{G}\left(t_{i}\right)-G_{M}\left(t_{i},g\right)\right]}^{2}}{{\left[\bar{G}\left(t_{i}\right)\right]}^{2}}.$$

is $J_{N}\left(g^{*}\right)=0.00907$. The course of the optimal FMM $G_{M}\left(t,g^{*}\right)$ and the real characteristic *G(t)* (62) are summarized in Figure 13a, where the measurements $\bar{G}\left(t_{i}\right)$ of the real modulus G(t) (62) are marked. The relaxation time spectrum $ℋ\left(\tau ,g^{*}\right)$ (11) is plotted in Figure 13b, together with the spectrum (61) of the real material.

#### 3.9.2. Example 2

**Figure 14:**
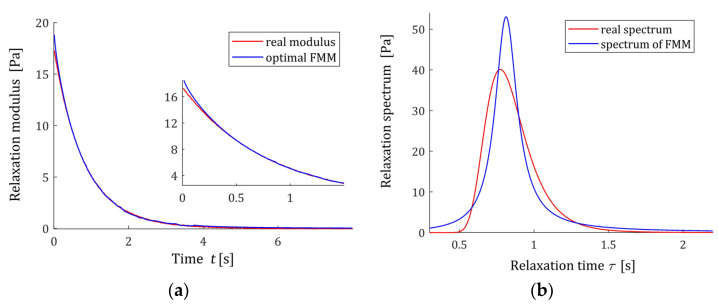
For the “real” material from Example 2 and the fractional Maxwell model (57) with optimal parameters $g^{*}$ (65) are presented: (**a**) the measurements $\bar{G}\left(t_{i}\right)$ of the real relaxation modulus G(t) (62) (red points) and model $G_{M}\left(t,g^{*}\;\right)$ (57); (**b**) real relaxation time spectrum ℋ(τ) (61) (solid red line) and the spectrum model $ℋ\left(\tau ,g^{*}\;\right)$ (11).

Now, the parameters of the Gauss-like distribution (61) are as follows: ϑ=31.52 Pa·s, $m=1.253\;s^{-1}$, and $q=9.73\times 10^{-2}{\;s}^{-2}$. In the experiment, N=1000 sampling instants $t_{i}$ were generated with the constant period in the time interval T=[0,8] seconds chosen in view of the course of the modulus G(t) (62). Additive measurement noises $z\left(t_{i}\right)$ were selected independently by random choice with uniform distribution on the interval [−0.05, 0.05] Pa. The optimal parameters of the model (57) are determined

(65) $$g^{*}={\left[\begin{array}{cccc} \alpha^{*} & \beta^{*} & E^{*} & \tau_{r}^{*} \end{array}\right]}^{T}={\left[\begin{array}{cccc} 0.983 & 0.017 & 17.77816\;\mathrm{Pa} & 0.81568\;s \end{array}\right]}^{T},$$

the optimal mean square identification index (59) is $Q_{N}\left(g^{*}\right)=1.6615788\times 10^{-2}\;\left[{\mathrm{Pa}}^{2}\right]$. The optimal FMM $G_{M}\left(t,g^{*}\right)$ and the real modulus G(t) (62) are plotted in Figure 14a. The relaxation time spectrum $ℋ\left(\tau ,g^{*}\;\right)$ (11) is plotted in Figure 14b, together with the real material spectrum (61).

### 3.10. Applicability of the FMM Spectra to Modelling BSW Spectra

**Figure 15:**
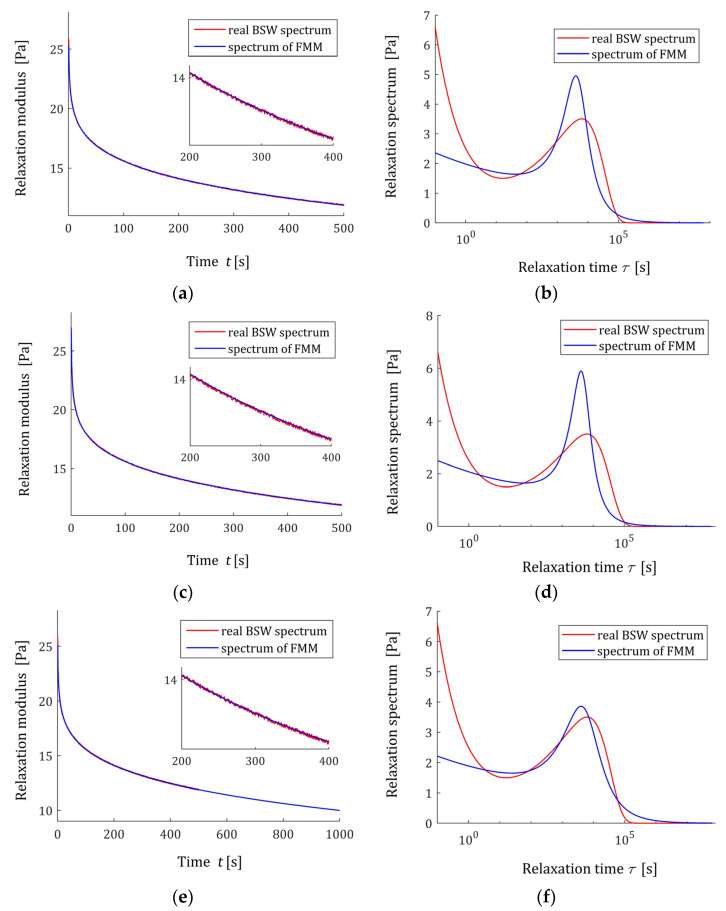
Relaxation modulus G(t) (red points) of the “real” material described by BSW spectrum ℋ(τ) (66) (solid red line) and the fractional Maxwell model $G_{M}\left(t,g^{*}\;\right)$ (57) and relaxation time spectra $ℋ\left(\tau ,g^{*}\;\right)$ (11), the model optimal parameters $g^{*}$ are given in Table 1 for: (**a**,**b**) experiment 1; (**c**,**d**) experiment 2; (**e**,**f**) experiment 3.

**Figure 16:**
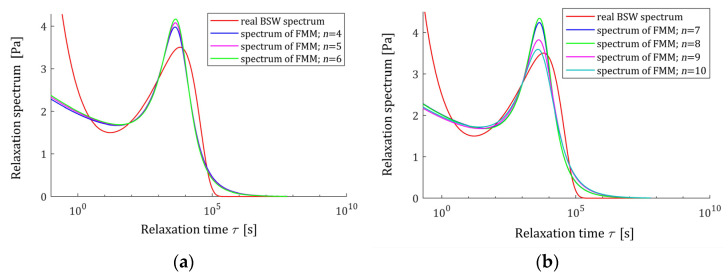
Relaxation spectra of the “real” material described by BSW spectrum ℋ(τ) (66) (solid red line) and relaxation time spectra $ℋ\left(\tau ,g^{*}\;\right)$ (11) of the fractional Maxwell model for numerical experiments: (**a**) n=4, 5, 6; (**b**) n=7, 8, 9, 10. The optimal parameters $g^{*}$ are given in Table 1.

Consider the spectrum of relaxation times introduced by Baumgaertel, Schausberger, and Winter [26,27],

(66) $$ℋ\left(\tau \right)=\left\{\beta_{1}{\left(\frac{\tau }{\tau_{c}}\right)}^{\rho_{1}}+\beta_{2}{\left(\frac{\tau }{\tau_{c}}\right)}^{\rho_{2}}\right\}e^{-\frac{\tau }{\tau_{max}}},$$

which is known to be effective in describing polydisperse polymer melts [28,29], with the parameters [29]: $\beta_{1}=6.276\times 10^{4}\;\mathrm{Pa}$, $\beta_{2}=1.27\times 10^{5}\;\mathrm{Pa}$, $\tau_{c}=2.481\;s$, $\tau_{max}=2.564\times 10^{4}\;s$, $\rho_{1}=0.25$ and $\rho_{2}=-0.5$. The corresponding ‘real’ relaxation modulus G(t) is given by (7). In the experiment, N time instants $t_{i}$ were sampled with the constant period in the time interval T=[0,T]. The results of the numerical experiment for several values of N and T are given in Table 1 and illustrated by Figure 12 and Figure 13. In Figure 15, the real material spectrum (66) along with the model $ℋ\left(\tau ,g^{*}\;\right)$ (11) and real modulus G(t) fitted by the optimal FMM $G_{M}\left(t,g^{*}\right)$, are plotted for the first three experiments. Since the fit of the model to the measurement data is very good and the waveforms of the characteristics for the relaxation modulus practically coincide with the measurement points and do not differ between each other, only the spectra are presented for three subsequent numerical experiments in Figure 16. These spectra also almost merge, however the maximum peak increases slightly with a growing number of measurement points N and decreases with increasing experiment time T.

## 4. Conclusions

Analytical formulas describing relaxation time and frequency spectra of FMM were given. The analytical studies proved that:

1. Necessary and sufficient conditions for the existence of the local extrema, minima, and maxima of the relaxation spectra are given by two algebraic inequalities.
2. Only two fractional model parameters, the non-integer orders of the stress and strain derivatives, uniquely determine the existence of the spectrum extrema.
3. The local minima and maxima of the relaxation spectra are described by direct analytical formulas.
4. The local extrema depend on fractional model parameters and on the relaxation time of FMM.
5. The spectrum values for the local extrema are affected by the elastic modulus of FMM, i.e., by all four model parameters.

Analytical analysis combined with numerical studies of model monotonicity and the spectra applicability to modelling BSW and Gauss-like spectra demonstrated that the viscoelastic relaxation spectrum of FMM can be applied for a wide class of polymers with unimodal relaxation spectra. This is due to the availability of the two extra degrees of freedom, non-integer orders of the stress and strain derivatives, which provides deep insight into the complex behavior of polymers.

The applicability of the relaxation spectra of fractional order viscoelastic models to the description of multimodal spectra will be the subject of future research, with particular attention to bimodal spectra that characterize many polymers. A respective modification of the fractional Maxwell model is then necessary. Since the identification of FMM is, in general, difficult, mainly due to the form of the relaxation modulus model form given by the product of Mittag-Leffler and power functions, approximate identification methods are still needed. Future research will be focused on this issue. Multi-scale combined power-law Scott-Blair model or FMM is a dilemma that may accompany the modelling of polymers governed by power-laws. It sets another research direction in the field of fractional viscoelasticity of polymers.

## Figures and Tables

**Table 1:** The parameters of the optimal models for the BSW spectrum ℋ(τ) (66) (62) in successive numerical experiments: number of numerical experiment n, number of measurements N, the time horizon of the experiment T, mean quadratic identification index $Q_{N}\left(g^{*}\right)$ (59), and mean relative quadratic identification index $J_{N}\left(g^{*}\right)$ (64), and the optimal FMM parameters $\alpha^{*}$, $\beta^{*}$, $E^{*}$, and $\tau_{r}^{*}$.

n	N	T [s]	$Q_{N}\left(g^{*}\right)\;\left[Pa^{2}\right]$	$J_{N}\left(g^{*}\right)$	$\alpha^{*}$	$\beta^{*}$	$E^{*}\;\left[Pa\right]$	$\tau_{r}^{*}\;\;\left[s\right]$
1	1000	500	1.5247 × 10^−3^	5.9182 × 10^−6^	0.802746	0.078769	12.89118	5.0226 × 10^3^
2	2000	500	2.4712 × 10^−3^	7.8212 × 10^−6^	0.853718	0.08208	12.67277	4.8057 × 10^3^
3	2000	1000	1.3271 × 10^−3^	6.8742 × 10^−6^	0.716114	0.07468	13.16061	5.7473 × 10^3^
4	3000	1000	1.6420 × 10^−3^	7.6078 × 10^−6^	0.731290	0.07648	13.00037	5.8162 × 10^3^
5	4000	1000	1.9309 × 10^−3^	8.1768 × 10^−6^	0.74252	0.07774	12.89206	5.8528 × 10^3^
6	5000	1000	2.2048 × 10^−3^	8.72088 × 10^−6^	0.751992	0.07872	12.80960	5.8698 × 10^3^
7	6000	1000	2.4493 × 10^−3^	9.08398 × 10^−6^	0.760015	0.07951	12.74484	5.8762 × 10^3^
8	7000	1000	2.7029 × 10^−3^	9.56398 × 10^−6^	0.765044	0.08008	12.69891	5.8907 × 10^3^
9	7000	1500	1.7812 × 10^−3^	8.9981 × 10^−6^	0.717188	0.07632	13.01787	5.9833 × 10^3^
10	7000	2000	1.3794 × 10^−3^	9.2961 × 10^−6^	0.700429	0.074402	13.19768	5.9004 × 10^3^

## Data Availability

Not applicable.

## Appendix A

### Appendix A.1. Derivation of Proposition 1

Consider spectral representation (9), (10) of the Mittag-Leffler function. Let us put μ=1−β and κ=α−β. By the assumptions 0<β<α≤1, the parameter 0<κ<1 and, simultaneously, parameter μ=1−β<1+κ. For real $z=-{\left(\frac{t}{\tau_{r}}\right)}^{\alpha -\beta }$ the argument |arg(z)|=π>πκ, thus the formula (9) holds and take the form

(A1) $$E_{\alpha -\beta ,1-\beta }\left(-{\left(\frac{t}{\tau_{r}}\right)}^{\alpha -\beta }\right)=\int_{0}^{\infty }K\left(\alpha -\beta ,1-\beta ,r,-{\left(\frac{t}{\tau_{r}}\right)}^{\alpha -\beta }\right)dr,$$

with

(A2) $$K\left(\alpha -\beta ,1-\beta ,r,-{\left(\frac{t}{\tau_{r}}\right)}^{\alpha -\beta }\right)=\frac{1}{\pi \left(\alpha -\beta \right)}r^{\frac{\beta }{\alpha -\beta }}e^{-r^{\frac{1}{\alpha -\beta }}}\frac{r\sin\left(\pi \beta \right)+{\left(\frac{t}{\tau_{r}}\right)}^{\alpha -\beta }\sin\left(\pi \alpha \right)}{r^{2}+2r{\left(\frac{t}{\tau_{r}}\right)}^{\alpha -\beta }\cos\left[\pi \left(\alpha -\beta \right)\right]+{\left(\frac{t}{\tau_{r}}\right)}^{2\left(\alpha -\beta \right)}}.$$

In view of (A2), the integral on the right-hand side of (A1) takes the form

$$E_{\alpha -\beta ,1-\beta }\left(-{\left(\frac{t}{\tau_{r}}\right)}^{\alpha -\beta }\right)=\frac{1}{\pi \left(\alpha -\beta \right)}\int_{0}^{\infty }r^{\frac{\beta }{\alpha -\beta }}e^{-r^{\frac{1}{\alpha -\beta }}}\frac{r\sin\left(\pi \beta \right)+{\left(\frac{t}{\tau_{r}}\right)}^{\alpha -\beta }\sin\left(\pi \alpha \right)}{r^{2}+2r{\left(\frac{t}{\tau_{r}}\right)}^{\alpha -\beta }\cos\left[\pi \left(\alpha -\beta \right)\right]+{\left(\frac{t}{\tau_{r}}\right)}^{2\left(\alpha -\beta \right)}}dr,$$

whence, by applying the substitution $r^{\frac{1}{\alpha -\beta }}=tv$, after algebraic manipulations we obtain

(A3) $$E_{\alpha -\beta ,1-\beta }\left(-{\left(\frac{t}{\tau_{r}}\right)}^{\alpha -\beta }\right)=\frac{\tau_{r}^{\alpha }}{\pi }{\left(\frac{t}{\tau_{r}}\right)}^{\beta }\int_{0}^{\infty }\frac{{\left(\tau_{r}v\right)}^{\alpha -\beta }\sin\left(\pi \beta \right)+\sin\left(\pi \alpha \right)}{{\left(\tau_{r}v\right)}^{2\left(\alpha -\beta \right)}+2{\left(\tau_{r}v\right)}^{\alpha -\beta }\cos\left[\pi \left(\alpha -\beta \right)\right]+1}v^{\alpha -1}e^{-tv}dv.$$

Combining (5), (A1) and (A3) yield

$$G\left(t\right)=E\frac{\tau_{r}^{\alpha }}{\pi }\int_{0}^{\infty }\frac{{\left(\tau_{r}v\right)}^{\alpha -\beta }\sin\left(\pi \beta \right)+\sin\left(\pi \alpha \right)}{{\left(\tau_{r}v\right)}^{2\left(\alpha -\beta \right)}+2{\left(\tau_{r}v\right)}^{\alpha -\beta }\cos\left[\pi \left(\alpha -\beta \right)\right]+1}v^{\alpha -1}e^{-tv}dv,$$

whence, by virtue of (8) model, (13) results. By the relation $ℋ\left(\tau \right)=H\left(\frac{1}{\tau }\right)$, we immediately obtain Equation (12), which can be rewritten as formula (11); proposition is derived. □

### Appendix A.2. Derivation of Proposition 2

Since all exponents in powers of the expression $\left(\tau_{r}v\right)$ in (13) are positive, the first boundary condition (17) is obvious. The spectrum (13) can be equivalently expressed as

(A4) $$H\left(v\right)=\frac{E}{\pi }\cdot \frac{{\left(\tau_{r}v\right)}^{\beta }\sin\left(\pi \beta \right)+\sin\left(\pi \alpha \right){\left(\tau_{r}v\right)}^{2\beta -\alpha }}{1+2{\left(\tau_{r}v\right)}^{-\left(\alpha -\beta \right)}\cos\left[\pi \left(\alpha -\beta \right)\right]+{\left(\tau_{r}v\right)}^{-2\left(\alpha -\beta \right)}}\;,$$

where the denominator of the right-hand side tends to 1, as v→∞, while the nominator tends to infinity regardless of the sign of the expression 2β−α, as v→∞, whence the second boundary condition (18) follows. By the relation $ℋ\left(\tau \right)=H\left(\frac{1}{\tau }\right)$, (19) and (20) directly follow from (18) and (17), respectively. □

### Appendix A.3. Proof of Property 1

The analysis of the spectra monotonicity is based on the formulas (12) and (13) describing ℋ(τ) and H(v), respectively. It is convenient to express these functions in equivalent, joint and more useful for further analysis form. Let us introduce the function

(A5) $$\varphi \left(x\right)=x^{\frac{\beta }{\alpha -\beta }}\frac{c_{1}x^{2}+c_{2}x}{x^{2}+2c_{3}x+1}\;,$$

where the coefficients $c_{1}$, $c_{2}$, and $c_{3}$ are defined by (21), (22), and (23). For

(A6) $$x={\left(\tau_{r}v\right)}^{\alpha -\beta },$$

by (13), (A5), and (A6), we have

(A7) $$H\left(v\right)={\frac{E}{\pi }\varphi \left(x\right)|}_{x={\left(\tau_{r}v\right)}^{\alpha -\beta }}\;.$$

If

(A8) $$x={\left(\frac{\tau_{r}}{\tau }\right)}^{\alpha -\beta },$$

then by (A5) and (12)

(A9) $$ℋ\left(\tau \right)={\frac{E}{\pi }\varphi \left(x\right)|}_{x={\left(\frac{\tau_{r}}{\tau }\right)}^{\alpha -\beta }}\;.$$

By (A7)

(A10) $$\frac{dH\left(v\right)}{dv}={\frac{\left(\alpha -\beta \right)E\tau_{r}}{\pi }{\left(\tau_{r}v\right)}^{\alpha -\beta -1}\frac{d\varphi \left(x\right)}{dx}|}_{x={\left(\tau_{r}v\right)}^{\alpha -\beta }}\;,$$

while by (A9)

(A11) $$\frac{dℋ\left(\tau \right)}{d\tau }=-{\frac{\left(\alpha -\beta \right)E}{\pi \tau_{r}}{\left(\frac{\tau_{r}}{\tau }\right)}^{\alpha -\beta +1}\frac{d\varphi \left(x\right)}{dx}|}_{x={\left(\frac{\tau_{r}}{\tau }\right)}^{\alpha -\beta }}\;.$$

The comparison of the two above formulas directly implies Property 1. □

### Appendix A.4. Proof of Proposition 3

In view of (A10) and (A11), to study the monotonicity of the spectra H(v) and ℋ(τ) for positive arguments, it is enough to analyze the monotonicity of φ(x) (A5). The first derivative of φ(x) (A5) is as follows

(A12) $$\frac{d\varphi \left(x\right)}{dx}=\frac{\frac{1}{\alpha -\beta }\;\;x^{\frac{\beta }{\alpha -\beta }}\;\psi \left(x\right)}{{\left[x^{2}+2c_{3}x+1\right]}^{2}},$$

where the function ψ(x) in nominator is given by

$$\psi \left(x\right)=\beta c_{1}x^{3}+\left[\left(2\beta -\alpha \right)c_{2}+2\alpha c_{1}c_{3}\right]x^{2}+\left[\left(2\alpha -\beta \right)c_{1}+2\beta c_{2}c_{3}\right]x+\alpha c_{2},$$

and can be expressed as

(A13) $$\psi \left(x\right)=\beta c_{1}x^{3}+c_{4}x^{2}+c_{5}x+\alpha c_{2},$$

with the coefficients $c_{4}$ and $c_{5}$ defined by (24) and (25), respectively. Since, under the assumptions 0<β<α≤1, coefficients $\beta c_{1}$ and $c_{5}$ are positive and $\alpha c_{2}\geq 0$, then inequality $c_{4}<0$ is necessary, but not sufficient, for the existence of the local extreme of φ(x); Proposition 3 follows. □

### Appendix A.5. Introduction to the Necessary and Sufficient Extrema Conditions

The properties of the cubic function ψ(x) (A13) and in consequence the monotonicity of the relaxation spectrum depends on the relationship between the parameters α and *β*. Since the denominator on the right-hand side of (A12) and the multiplier $x^{\frac{\beta }{\alpha -\beta }}$ in (A12) are positive for all x>0, both the sign of the derivative dφ(x)/dx and their nonzero real roots, if they exist, are identical to those of ψ(x). Thus, in view of (A10), (A11), and (A12), function φ(x) and in consequence the relaxation spectra H(v) and ℋ(τ) have local extrema, the local maximum for the relaxation frequency $v=v_{max}>0$ and the local minimum for the minimum frequency $v=v_{min}>0$, if and only if the respective $x_{max}={\left(\tau_{r}v_{max}\right)}^{\alpha -\beta }>0$ (c.f., (A6)) and $x_{min}={\left(\tau_{r}v_{min}\right)}^{\alpha -\beta }>0$ are the roots of the cubic function ψ(x) (A13). Thus, the existence, or not, of two positive real roots of the function ψ(x) is basic for the spectrum monotonicity.

Due to $\beta c_{1}>0$, $\underset{x\to \infty }{\lim}\psi \left(x\right)=+\infty$ and $\underset{x\to -\infty }{\lim}\psi \left(x\right)=-\infty$. Simultaneously, $\psi \left(0\right)=\alpha c_{2}>0$ whenever α<1 and $\psi \left(0\right)=\alpha c_{2}=0$ for α=1. Thus, in further analysis, two different cases should be distinguished, when α is equal to one, or not. □

### Appendix A.6. Proof of Proposition 4

If parameter α<1, then the cubic function ψ(x) has at least one real root on the negative real axis. The necessary and sufficient conditions of the existence of three real roots of third order polynomials are known, as well as the analytical methods for their computation. The algebraic solution of the cubic equation can be derived in a number of different ways. Cardano’s method, dated 1545, and Vieta’s method published in 1615 are the most known. The two methods are combined here and applied to the cubic equation ψ(x)=0, which, in view of (A13), takes the form:

(A14) $$\beta c_{1}x^{3}+c_{4}x^{2}+c_{5}x+\alpha c_{2}=0.$$

Dividing Equation (A14) by the coefficient $\beta c_{1}$ and applying the standard substitution

(A15) $$x=z-\frac{c_{4}}{3\beta c_{1}},$$

we obtain the so-called depressed cubic equation with the zero quadratic term coefficient:

(A16) $$z^{3}+3pz+2q=0,$$

where the parameters p and q are such that

(A17) $$3p=\frac{3\beta c_{1}c_{5}-c_{4}^{2}}{3{\left[\beta c_{1}\right]}^{2}},$$

(A18) $$2q=\frac{2c_{4}^{3}}{27{\left[\beta c_{1}\right]}^{3}}-\frac{c_{4}c_{5}}{3{\left[\beta c_{1}\right]}^{2}}+\frac{\alpha c_{2}}{\beta c_{1}}.$$

The number and types of the roots are uniquely determined by the determinant of the cubic equation defined as follows

(A19) $$D=q^{2}+p^{3}.$$

The depressed cubic Equation (A16) has three real roots if and only if D≤0. Thus, if D>0, then ψ(x) and also derivative $\frac{d\varphi \left(x\right)}{dx}$ (A12) is positive for all x>0. Therefore, spectrum H(v) is a monotonically increasing function, and spectrum ℋ(τ) decreases with increasing τ.

Let us analyze two cases in detail: (a) D=0, (b) D<0.

#### Appendix A.6.1. Case (a). The Determinant D=0

If the determinant D=0, then Equation (A16) and consequently (A14) have multiple real roots, all of their roots are real.

If p=q=0, i.e., $3\beta c_{1}c_{5}=c_{4}^{2}$ and $2c_{4}^{3}-9c_{4}c_{5}\beta c_{1}+27\alpha c_{2}{\left[\beta c_{1}\right]}^{2}=0$, which is equivalent to $9\alpha \beta c_{1}c_{2}=c_{4}c_{5}$ and implies $c_{4}c_{5}>0$, the triple root is such that $x_{1\_3}=-\frac{c_{4}}{3\beta c_{1}}=-\frac{c_{5}}{c_{4}}<0$; for derivation, Equation (A20) given below may be used. Thus, ψ(x)>0 for all x>0, spectra H(v) and ℋ(τ) are monotonically increasing and decreasing functions.

If $p^{3}=-q^{2}\neq 0$, then Equation (A16) has two real roots, and one of them is double. It may be proved that single root is negative. Even if the double root is positive, the function ψ(x), whence also derivative $\frac{d\varphi \left(x\right)}{dx}$ (A12), is positive on both sides of the root, being an inflection point (i.e., saddle point) of the function φ(x). Thus, the respective relaxation frequency and time are the inflection points of the monotonically increasing and decreasing spectra H(v) and ℋ(τ), respectively. Combining the spectra monotonicity for D=0, with the monotonicity in case D>0 yields Proposition 4 with inequality (30), resulting directly from (A19), (A17), and (A18).

#### Appendix A.6.2. Case (b). The Determinant D<0

If D<0, then the cubic Equation (A16) roots are obtained by Viète’s formulas [59] in terms of trigonometric functions (except when p=0, but it is not the case for D<0), which, in view of (A15) for the original third order Equation (A14), results in the three different real roots:

(A20) $$x_{1}=-2r\cos\left(\frac{\theta }{3}\right)-\frac{c_{4}}{3\beta c_{1}},$$

(A21) $$x_{2}=2r\cos\left(\frac{\pi -\theta }{3}\right)-\frac{c_{4}}{3\beta c_{1}},$$

(A22) $$x_{3}=2r\cos\left(\frac{\pi +\theta }{3}\right)-\frac{c_{4}}{3\beta c_{1}},$$

where

(A23) $$r=sgn\left(q\right)\sqrt{\left|p\right|},$$

and the angle θ is such that

(A24) $$cos\left(\theta \right)=\frac{q}{r^{3}}.$$

The condition D<0, in view of (A19), implies p<0, and next

$$0\leq \left|q\right|<\left|p\right|\sqrt{\left|p\right|},$$

whence, by (A24), the next inequality results

$$0\leq cos\left(\theta \right)=\frac{q}{sgn\left(q\right)\left|p\right|\sqrt{\left|p\right|}}=\frac{\left|q\right|}{\left|p\right|\sqrt{\left|p\right|}}<1.$$

Thus, the angle *ɵ* is such that

$$0<\theta =arc\;cos\left(\frac{q}{r^{3}}\right)\leq \frac{\pi }{2}.$$

If q>0, then by (A23), r>0 and the inequalities occur

$$-2r\cos\left(\frac{\theta }{3}\right)<0<2r\cos\left(\frac{\pi +\theta }{3}\right)<2r\cos\left(\frac{\pi -\theta }{3}\right),$$

whence, in view of (A20), (A21), (A22), having in mind the monotonicity of ψ(x) and the assumption $c_{4}<0$, we conclude that

$$x_{1}<0<x_{3}<x_{2}.$$

If q<0, then r<0 and we have the inequalities

$$-2r\cos\left(\frac{\theta }{3}\right)>0>2r\cos\left(\frac{\pi +\theta }{3}\right)>2r\cos\left(\frac{\pi -\theta }{3}\right),$$

whence, from the assumption $c_{4}<0$ and monotonicity of ψ(x), the relation results

$$x_{1}>x_{3}>0>x_{2}.$$

If q=0, then r>0 and, by (A24), cos(θ)=0, whence $\theta =\frac{\pi }{2}$ and, by $c_{4}<0$, the three real roots are such that

$$x_{1}<0<x_{3}<x_{2}.$$

Proposition 4 has been proved, where inequality (29) means that the determinant D<0 simply results from (A17), (A18), and (A19). □

### Appendix A.7. Derivation of Proposition 5

If the relaxation frequency (13) and time (11) spectra have extrema, then inequality (29) holds, i.e., in particular the second summand of its left-hand side is negative, whence inequality (33) results. By standard trigonometric identities, we have

$$c_{4}^{2}={\left[2\beta \sin\left(\pi \alpha \right)+\alpha \sin\left[\pi \left(2\beta -\alpha \right)\right]\right]}^{2}={\left[2\beta \sin\left(\pi \alpha \right)\right]}^{2}+{\left[\alpha \sin\left[\pi \left(2\beta -\alpha \right)\right]\right]}^{2}+4\beta \sin\left(\pi \alpha \right)\alpha \sin\left[\pi \left(2\beta -\alpha \right)\right],$$

and next

(A25) $$c_{4}^{2}=2\beta^{2}\left[1-\cos\left(2\pi \alpha \right)\right]+\frac{1}{2}\alpha^{2}\left[1-\cos\left(2\pi \left(2\beta -\alpha \right)\right)\right]+\;2\beta \cos\left(2\pi \left(\alpha -\beta \right)\right)-2\beta \cos\left(2\pi \beta \right).$$

By (21) and (27)

$$c_{1}c_{5}=2\alpha \sin\left(\pi \beta \right)\sin\left(\pi \beta \right)+\beta \sin\left(\pi \beta \right)\sin\left[\pi \left(2\alpha -\beta \right)\right],$$

which can be expressed as

(A26) $$c_{1}c_{5}=\alpha \left[1-\cos\left(2\pi \beta \right)\right]+\frac{1}{2}\beta \cos\left[2\pi \left(\beta -\alpha \right)\right]-\frac{1}{2}\beta \cos(2\pi \alpha ).$$

Combining (A26), (A25), and (33), after algebraic manipulations, yields

$$-\beta \alpha cos\left(2\pi \beta \right)+\frac{3}{2}\beta^{2}\cos\left[2\pi \left(\beta -\alpha \right)\right]<-\frac{1}{2}\beta^{2}\cos\left(2\pi \alpha \right)+\frac{1}{2}\alpha^{2}+2\beta^{2}-3\beta \alpha -\frac{1}{2}\alpha^{2}\cos\left[2\pi \left(2\beta -\alpha \right)\right]+2\beta \cos\left[2\pi \left(\alpha -\beta \right)\right].$$

Hence, by virtue of the parity of the cosine function, we obtain

$$-\beta \alpha cos\left(2\pi \beta \right)<-\frac{1}{2}\beta^{2}\cos\left(2\pi \alpha \right)+\frac{1}{2}\alpha^{2}+2\beta^{2}-3\beta \alpha -\frac{1}{2}\alpha^{2}\cos\left[2\pi \left(2\beta -\alpha \right)\right]+\left(2\beta -\frac{3}{2}\beta^{2}\right)\cos\left[2\pi \left(\alpha -\beta \right)\right],$$

which is equivalent to (34). The proposition is proved. □

### Appendix A.8. Proof of Proposition 7

Now, by (48) and (49), function φ(x) (A5), due to (A7) and (A9) important for an analysis of the spectrum monotonicity, takes a simple form described by

(A27) $$\varphi \left(x\right)=x^{\frac{\beta }{1-\beta }}\frac{c_{1}x^{2}}{x^{2}-2c_{6}x+1}\;,$$

with the argument x given by (A6) and (A8), where parameter

$$c_{6}=\cos\left(\pi \beta \right).$$

From (A27), in straightforward way, we have

$$\frac{d\varphi \left(x\right)}{dx}=\frac{c_{1}}{1-\beta }x^{\frac{1}{1-\beta }}\frac{\psi_{1}\left(x\right)}{{\left[x^{2}-2c_{6}x+1\right]}^{2}},$$

where the function $\psi_{1}\left(x\right)$ in the nominator is as follows

$$\psi_{1}\left(x\right)=\beta x^{2}-2c_{6}x+2-\beta .$$

Since $\psi_{1}\left(0\right)=2-\beta >0$, the stationary point equation $\frac{d\varphi \left(x\right)}{dx}=0$ has for x>0 two positive real roots, if and only if the determinant

(A28) $$\Delta_{1}=4c_{6}^{2}-4\beta \left(2-\beta \right)=4\left[\cos^{2}\left(\pi \beta \right)-\beta \left(2-\beta \right)\right],$$

of the square equation $\psi_{1}\left(x\right)=0$ is nonnegative, i.e.,

(A29) $$\cos^{2}\left(\pi \beta \right)\geq \beta \left(2-\beta \right),$$

and, simultaneously, $\left(2c_{6}-\sqrt{\Delta_{1}}\right)>0$, which, due to (A28), is equivalent to

(A30) $$\cos\left(\pi \beta \right)>\sqrt{\cos^{2}\left(\pi \beta \right)-\beta \left(2-\beta \right)}.$$

Then,

(A31) $$x_{1}=\frac{2c_{6}-\sqrt{\Delta_{1}}}{2\beta }=\frac{\cos\left(\pi \beta \right)-\sqrt{\cos^{2}\left(\pi \beta \right)-\beta \left(2-\beta \right)}}{\beta },$$

corresponds to $v_{max}$, while $v_{min}$ is determined by

(A32) $$x_{2}=\frac{2c_{6}+\sqrt{\Delta_{1}}}{2\beta }=\frac{\cos\left(\pi \beta \right)+\sqrt{\cos^{2}\left(\pi \beta \right)-\beta \left(2-\beta \right)}}{\beta }.$$

In the special case, when $\Delta_{1}=0$ and $\cos\left(\pi \beta \right)=\sqrt{\beta \left(2-\beta \right)}>0$, the double root $x_{1}=x_{2}=\frac{\cos\left(\pi \beta \right)}{\beta }=\sqrt{\frac{2-\beta }{\beta }}$ is the inflection point of the increasing function φ(x), thus spectrum H(v) (13) increases for all v>0, while ℋ(τ) is a monotonically decreasing function. Thus, bearing in mind inequality (A29) corresponding to $\Delta_{1}\geq 0$, the local extrema there exist, if and only if inequalities

(A33) $$\cos^{2}\left(\pi \beta \right)>\beta \left(2-\beta \right),$$

and (A30) are satisfied. Inequality (A33) is rewritten as (50). The fulfilment of inequality (50) implies the fulfilment of (A30). Bearing in mind Property 1 and the previous analysis, formulas (51), (53), (54), and (52) result directly from (A31) and (A32). Proposition 7 is proved. □
